# Functional loss of *Ccdc151* leads to hydrocephalus in a mouse model of primary ciliary dyskinesia

**DOI:** 10.1242/dmm.038489

**Published:** 2019-08-02

**Authors:** Francesco Chiani, Tiziana Orsini, Alessia Gambadoro, Miriam Pasquini, Sabrina Putti, Maurizio Cirilli, Olga Ermakova, Glauco P. Tocchini-Valentini

**Affiliations:** 1 European Mouse Mutant Archive (EMMA), INFRAFRONTIER, Monterotondo Mouse Clinic, Department of Biomedical Sciences (DSB), Italian National Research Council (CNR), Adriano Buzzati-Traverso Campus, via Ramarini, 32, 00015, Monterotondo, Rome, Italy; 2 Institute of Biochemistry and Cell Biology (IBBC), Department of Biomedical Sciences (DSB), Italian National Research Council (CNR), Adriano Buzzati-Traverso Campus, via Ramarini, 32, 00015, Monterotondo, Rome, Italy

**Keywords:** Gene knockout, X-ray gene expression imaging, MicroCT brain imaging, CSF, Cilia, IMPC

## Abstract

Primary ciliary dyskinesia (PCD) is a genetically heterogeneous disorder affecting normal structure and function of motile cilia, phenotypically manifested as chronic respiratory infections, laterality defects and infertility. Autosomal recessive mutations in genes encoding for different components of the ciliary axoneme have been associated with PCD in humans and in model organisms. The *CCDC151* gene encodes for a coiled-coil axonemal protein that ensures correct attachment of outer dynein arm (ODA) complexes to microtubules. A correct arrangement of dynein arm complexes is required to provide the proper mechanical force necessary for cilia beat. Loss-of-function mutations in *CCDC151* in humans leads to PCD disease with respiratory distress and defective left-right body asymmetry. In mice with the *Ccdc151^Snbl^* loss-of-function mutation (*Snowball* mutant), left-right body asymmetry with heart defects have been observed. Here, we demonstrate that loss of *Ccdc151* gene function via targeted gene deletion in mice leads to perinatal lethality and congenital hydrocephalus. Microcomputed tomography (microCT) X-ray imaging of Ccdc151–β-galactosidase reporter expression in whole-mount brain and histological analysis show that *Ccdc151* is expressed in ependymal cells lining the ventricular brain system, further confirming the role of *Ccdc151* dysfunction in hydrocephalus development. Analyzing the features of hydrocephalus in the *Ccdc151*-knockout animals by microCT volumetric imaging, we observe continuity of the aqueduct of Sylvius, indicating the communicating nature of hydrocephalus in the *Ccdc151*-knockout animals. Congenital defects in left-right asymmetry and male infertility have been also observed in *Ccdc151-*null animals. *Ccdc151* gene deletion in adult animals results in abnormal sperm counts and defective sperm motility.

This article has an associated First Person interview with the joint first authors of the paper.

## Abstract (precis)

**Summary:** *Ccdc151*-knockout mice develop PCD with hydrocephalus, situs abnormalities and male infertility. Novel 3D microCT imaging analysis of the *Ccdc151-lacZ* reporter gene demonstrates *Ccdc151* expression in ependymal cells.

## INTRODUCTION

Motile cilia play a critical role in the flow of physiological fluid along the epithelial surfaces in many organs and systems, both in human and mouse. In the nervous system cilia are essential for the normal flow of cerebrospinal fluid (CSF) within the ventricular brain system into the central canal of the spinal cord, while in respiratory and auditory systems cilia are required for mucus and fluid clearance (Mitchison and Valente, 2017; Bustamante-Marin and Ostrowski, 2017). Oviductal egg and sperm migration, sperm motility and directional flow of morphogens during early embryonic development that is necessary for correct left-right patterning also depend on proper ciliary function. In vertebrates, establishment of left-right body asymmetry is directed by leftward nodal flow created by directional ciliary beats across the vertebrate left-right organizer. This directional flow is necessary for induction of an asymmetric gene expression cascade in the lateral plate mesoderm, which determines left-right organ asymmetry (situs). Abnormalities in the direction or speed of the flow result in randomization of left-right body axis asymmetry (Yoshiba and Hamada, 2014; Sampaio et al., 2014).

Primary ciliary dyskinesia (PCD; OMIM:244400) is a complex disease caused by structural or developmental defects impeding normal function of motile cilia. Reported prevalence of PCD varies from 1:2000 to 1:40,000 people, indicative of a true variability among different populations, as well as difficulties in achieving a correct diagnosis (Kuehni and Lucas, 2016). Because motile cilia are essential for normal functioning of different physiological systems, PCD is a disorder affecting many organs. It is worth noting that all affected PCD patients share a common trait: respiratory tract infections, caused by impaired mucociliary clearance, which, in severe cases, can progress to lung destruction; the only exception is extremely light respiratory symptoms being observed in individuals carrying *DNAH9* mutation (Fassad et al., 2018). A subset of PCD patients have a defect in left-right body asymmetry, a condition defined as a Kartanger syndrome (Goutaki et al., 2019). Among those patients, slightly less than 50% show situs inversus and 10% carry other laterality defects such as situs ambiguus or heterotaxy (Shapiro et al., 2014). About 5% of PCD patients have simple or complex cardiovascular malformations (Goutaki et al., 2016). Male and female infertility is often reported in PCD patients (Ichioka et al., 2006; Eliasson et al., 1997). Other organs can be affected, and hydrocephalus, retinitis pigmentosa and renal cysts have been occasionally detected (Ibañez-Tallon et al., 2004; Afzelius, 2004; Knowles et al., 2013, 2016). Because of the heterogeneity in clinical manifestation of PCD, a PICADAR diagnostic predictive tool has been developed by the Swiss PCD Registry (CH-PCD) Working Group to facilitate correct PCD diagnosis based on clinical symptoms (Behan et al., 2016).

Congenital hydrocephalus, an abnormal accumulation of CSF in the brain ventricles, can be caused by abnormal production, absorption or flow of CSF within the ventricular brain system (McAllister, 2012; Perez-Figares et al., 2001). Anatomically it is characterized by ventricular enlargements due to CSF accumulation, damage of the underlying ependyma and thinning of the cerebral cortex. Unidirectional flow of the CSF from the third and fourth lateral ventricles into the spinal canal is driven by the coordinated movement of motile cilia found on ependymal cells lining the ventricular brain system. Abnormal CSF flow through the ventricle due to anomalous differentiation, migration or polarization of ependymal cells, as well as irregular direction or frequency of the ciliary beats or block in CSF ventricular circulation, can lead to congenital hydrocephalus in humans and mice (Jiménez et al., 2001; Crews et al., 2004; Lee, 2013; Fliegauf et al., 2007). Two types of hydrocephalus are generally recognized: (1) obstructive, when CSF flow in the ventricular brain system is physically blocked, and (2) communicating, which is caused by abnormal production, absorption or speed of CSF flow without interruption of continuity of the cerebral ventricular system (McAllister, 2012; Perez-Figares et al., 2001).

Motile cilia and sperm flagella are cellular organelles with axonemes built from microtubules and found on the surface of specialized cells. Each axoneme is composed of two central microtubules and nine peripheral microtubule doublets, which are connected to the central pair by radial spokes and enveloped by the ciliary membrane (Lodish et al., 2000). Specific components of motile cilia, which are necessary for cilia beatings, include inner dynein arms (IDAs) and outer dynein arms (ODAs), both of which are formed by dynein motor protein. Dyneins produce mechanical forces that enable microtubules to perform a coordinated sliding action against each other (Viswanadha et al., 2017). This is a very important part of the mechanism by which the rhythmic and synchronized cilia movements are produced. Multi-subunit dynein arm motor complexes are synthesized and preassembled in cytosol, transported to the cilium or flagella compartments, and anchored into the axonemal microtubule scaffold via the ODA-docking complex (ODA-DC) system. Around 70-80% of PCD cases are due to mutations in genes coding either for dynein arm proteins, ODA-DC components, or proteins responsible for assembly and transport of these complexes (Papon et al., 2010; Shoemark et al., 2012).

The *CCDC151* gene encodes for a coiled-coil protein critical for ODA-complex assembly. Nonsense mutations in all three coiled-coiled conserved domains of *CCDC151* patients were identified in seven human patients with PCD disease. All were suffering from respiratory infections and chronic sinusitis, while otitis media and a hearing defect were diagnosed for two of them. In addition, three patients had situs inversus totalis (SIT), two presented with cardiac ventricular septal defects, while the other two showed dextrocardia with situs inversus abdominalis (SIA) (Zhang et al., 2019; Hjeij et al., 2014; Alsaadi et al., 2014). Reconstitution biochemical experiments showed that the absence of functional CCDC151 impairs the binding of the DNAH5 protein to ODA complex, and absence of CCDC114 prevents ARMC4 proteins binding to ODA-DC (Hjeij et al., 2014). As a result, the complete loss of ODA from axonemes and severely impaired ciliary beating are observed. The role of Ccdc151 protein in ODA assembly and ciliary motility is conserved among vertebrates and confirmed both in *Drosophila* and zebrafish (Jerber et al., 2014). In mice, an N-ethyl-N-nitrosourea (ENU) mutagenesis screen for the congenital heart defects allowed discovery of a loss-of-function mutation in a splice donor site of the *Ccdc151* gene (*Snowball* mutation; *Ccdc151^Snbl^*; MGI: 5445347). These animals exhibited impaired tracheal and ependymal ciliary motility due to lack of ODAs. Consistent with impaired ciliary function, these mice presented with randomization of left-right asymmetry and heart malformation phenotypes (Hjeij et al., 2014; Li et al., 2015).

In this work, we demonstrate that the loss of *Ccdc151* gene function in mice leads to fast-progressing hydrocephalus and early postnatal lethality. We analyzed a *Ccdc151*-knockout mouse model in which *Ccdc151* exons 2 and 3 are replaced by a *lacZ* (encoding β-galactosidase) reporter gene. Using a *Ccdc151-lacZ* fusion gene and performing histological analysis, we show that the *Ccdc151* gene is expressed in ependymal cells of the brain. The results of this histological expression-pattern analysis were confirmed by *in vitro* direct three-dimensional (3D) X-ray imaging of the whole-mount brain by applying a novel in-house-developed experimental procedure based on the *in situ* detection of the β-galactosidase reaction products. Such a method allowed us to build the 3D pattern of *Ccdc151* gene expression in murine brain and to demonstrate that the *Ccdc151-lacZ* reporter is expressed in all anatomical compartments of the murine ventricular brain system. In agreement with the previously published data, we determined that the subset of *Ccdc151*-knockout animals also show laterality defects, such as SIT and SIA. We have also demonstrated that the *Ccdc151* gene is expressed in murine testes and is essential for normal sperm development. Complete azoospermia was observed in a few *Ccdc151-*null males, which occasionally survived post-weaning and reached sexual maturation. We also demonstrated that the conditional deletion of the *Ccdc151* gene in adult males causes low sperm counts and defective sperm motility. Overall, our work substantiates that the *Ccdc151* knockout is a valuable mouse model for the study of processes leading to human ciliopathies both during embryonic development and in adult animals.

## RESULTS

### Knockout of *Ccdc151* leads to early perinatal lethality in C57BL/6N mice

**Fig. 1.**
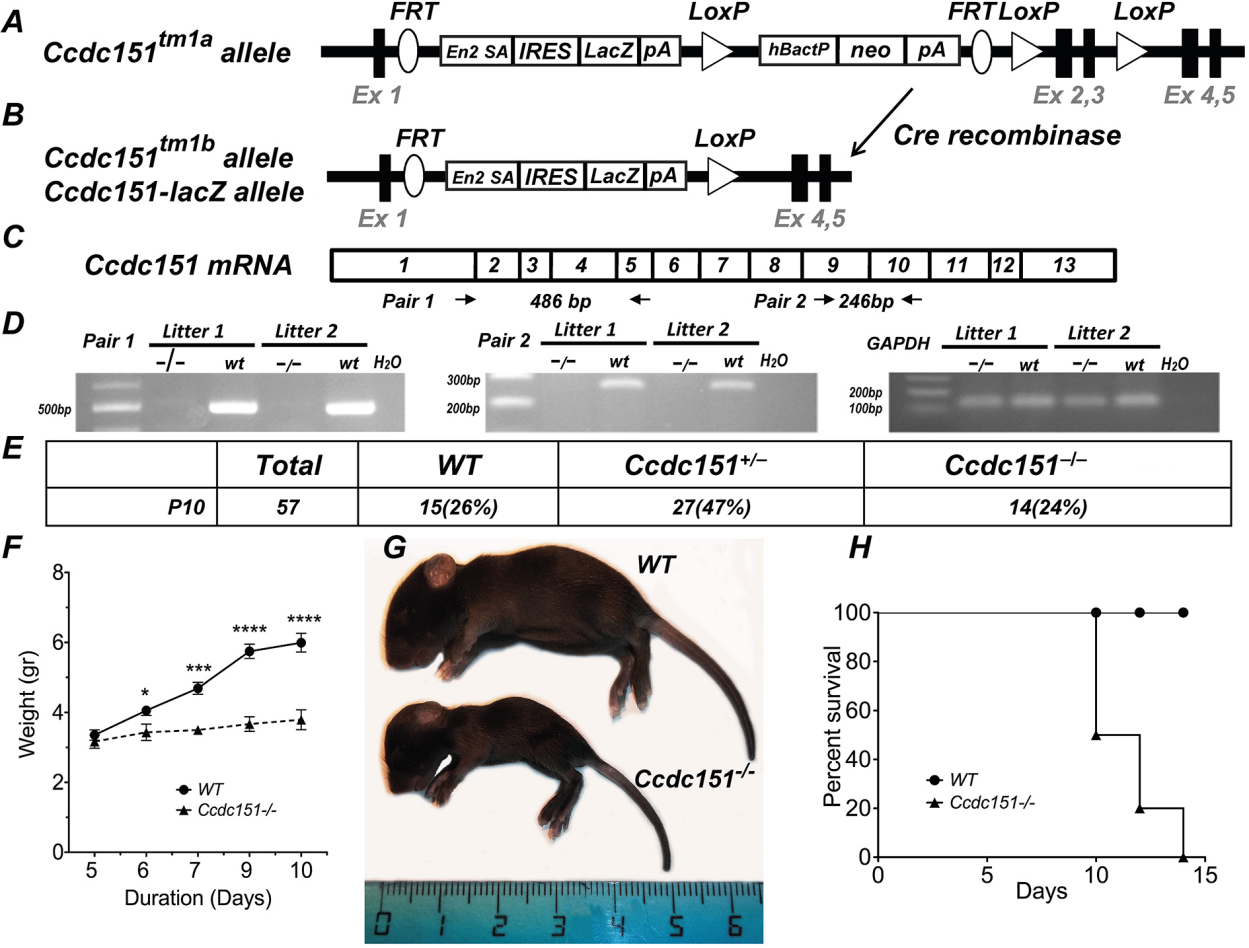
***Ccdc151* gene knockout leads to perinatal lethality in mice.** (A) Schematic representation of the *Ccdc151^t^^m1a^* allele. The *lacZ* reporter gene is inserted into an intronic locus, following exon 1, replacing exons 2 and 3 of the *Ccdc151* gene. (B) Schematic representation of the *Ccdc151^t^^m1b^* allele. The *Ccdc151^t^^m1b^* allele is generated from the *Ccdc151^t^^m1a^* allele by Cre-mediated recombination. Cre recombination deletes exons 2 and 3 of the *Ccdc151* gene and the *neo* targeting cassette from the *Ccdc151^t^^m1a^* genomic locus. The *Ccdc151^tm1^^b^* allele is a knockout of the *Ccdc151* gene and in the text is labeled as the *Ccdc151^−^* allele. Ex, exon; *En2A SA*, splice acceptor site; *IRES*, internal ribosomal entry site; *lacZ*, bacterial β-galactosidase reporter gene; *pA*, poly A; *hBactP*, human β-actin promoter; *neo*, neomycin resistance gene; *FRT*, FLP recombination sites; *LoxP*, Cre recombination sites. (C) Schematic representation of *Ccdc151* mRNA. Primers for the RT-PCR analysis, used to demonstrate the ablation *Ccdc151* gene expression, are indicated. (D) Loss of *Ccdc151* wild-type (WT) mRNA in homozygous *Ccdc151*-knockout animals is demonstrated by RT-PCR. (E) Analysis of genotype distribution of animals obtained by breeding between heterozygous *Ccdc151^+/−^* mice. Mendelian ratio of WT, *Ccdc151^+/−^* and *Ccdc151^−^*^/−^ animals is observed at postnatal day 10 (P10). (F) *Ccdc151*-knockout animals demonstrate severe growth retardation. Weight measurements were performed starting at P5. From P6, *Ccdc151^−^*^/−^ animals weighed significantly less when compared to WT animals (mean±s.e.m.; unpaired *t*-test; *P<0.05; ***P<0.001; ****P<0.0001). (G) Photograph of a *Ccdc151^−^*^/−^ animal and WT littermate. (H) Most of the *Ccdc151^−/−^* animals do not survive to weaning. Kaplan–Meier survival plot of the *Ccdc151-*null (*N*=10) and WT (*N*=10) animals; log-rank test (Chi square=13.13; d.f.=1; *P*=0.003).

To investigate the impact of loss of function of the *Ccdc151* gene, we engineered a mouse model in which the *Ccdc151* gene is deleted. Knockout mice were generated from *Ccdc151^tm1a(EUCOMM)Wtsi^* embryonic stem (ES) cells produced by the International Knockout Mouse Consortium (IKMC) (Fig. 1A). By breeding animals carrying the *Ccdc151^tm1a^* allele with *ROSA26Cre* (MGI: 5285392) animals, we produced the *Ccdc151^tm1b^* allele (MGI: 1924859; http://www.mousephenotype.org/data/genes/MGI:1924859). The *Ccdc151^t^^m1b^* allele is a knockout of the *Ccdc151* gene in which exons 2 and 3 are replaced by *lacZ* and a neomycin (*neo*) cassette that is present in the *Ccdc151^tm1a^* allele is removed from the targeted locus (Fig. 1B). This results in the expression of a *Ccdc151-lacZ* reporter gene and loss of endogenous *Ccdc151* mRNA due to out-of-frame mutation. We demonstrated the absence of wild-type *Ccdc151* mRNA by reverse transcription PCR (RT-PCR) using RNA extracted from the brain of homozygous *Ccdc151^tm1b/tm1b^* animals. Therefore, the *Ccdc151^tm1b^* allele is a null *Ccdc151* allele, which, in the text below, is denoted as the *Ccdc151**^−^*** allele (Fig. 1C,D).

We then investigated the consequences of the loss of *Ccdc151* in murine development. Heterozygous *Ccdc151*^+/−^ animal breedings produced, on average, litters of normal size with expected mendelian distribution of wild-type, *Ccdc151*^+/−^ and *Ccdc151*^−/−^ genotypes at postnatal day 10 (P10) (Fig. 1E). The first few days after birth the *Ccdc151^−/−^* pups were visually indistinguishable from their littermates. However, a statistically significant growth delay was observed already at P6 and increased dramatically at P10, with *Ccdc151^−/−^* being severely growth retarded at this point (Fig. 1F,G). In addition, all *Ccdc151^−/−^* animals presented with a dome-shaped head, a clear sign of hydrocephalus in mice (Fig. 1G). After the second postnatal week, most *Ccdc151^−/−^* littermates either died or had to be euthanized (Fig. 1H). Only a few *Ccdc151^−/−^* animals survived into the post-weaning period.

### *Ccdc151* is expressed in ependymal cells in murine brain

**Fig. 2.**
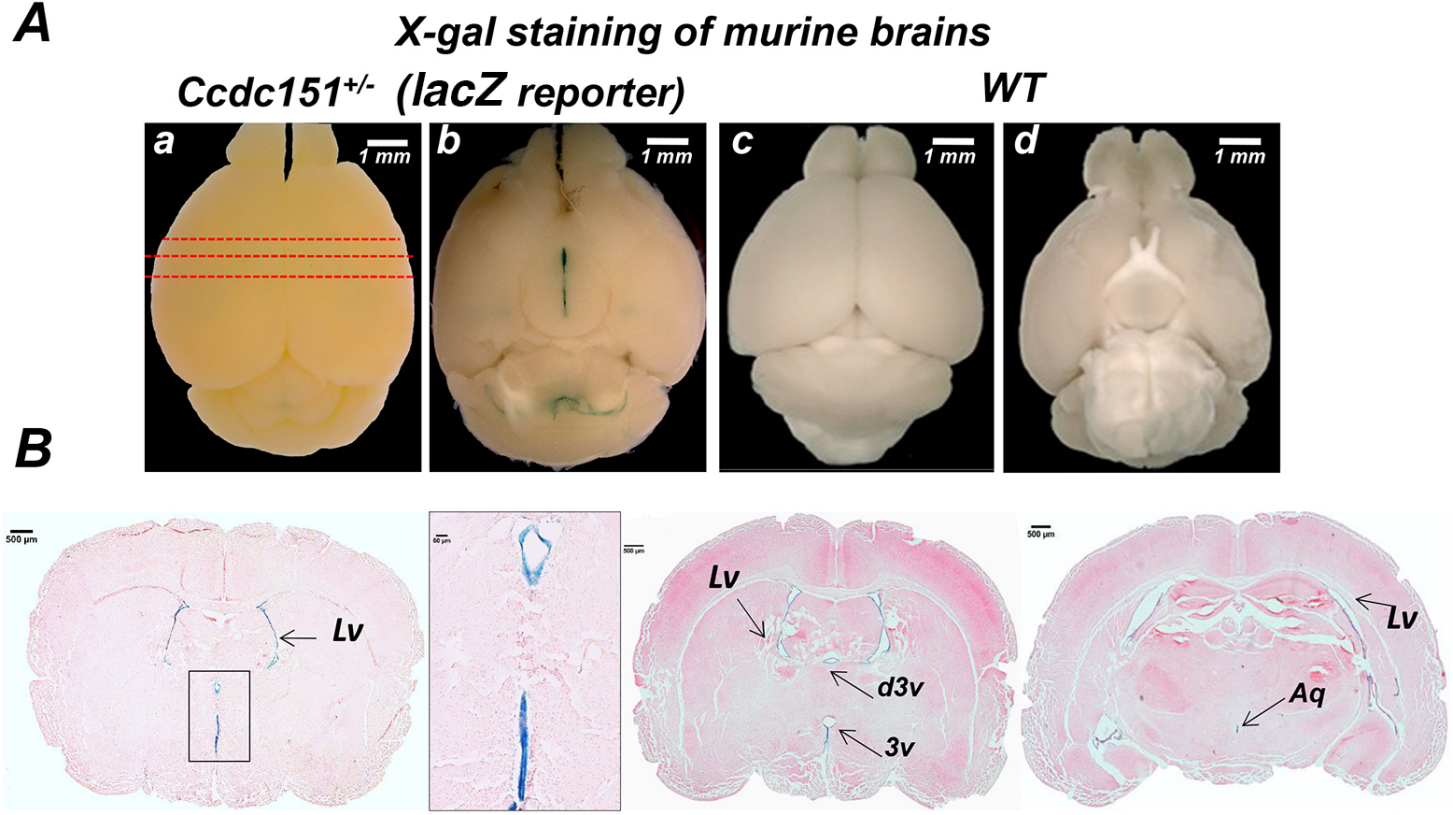
***Ccdc151* is expressed in ependymal cells of** **the** **ventricular brain system in mice.** (A) Dorsal and ventral views of the brains from *Ccdc151^+/−^* animals, expressing the *Ccdc151-lacZ* reporter gene (a,b), and wild-type animals (c,d)*.* The whole-mount brains were stained with X-gal/FeCN and β-galactosidase expression was visualized by steriomicroscopy. Red dashed lines indicate the sections of the brain presented in panel B. (B) Histological analysis of *Ccdc151* gene expression. Sections were obtained from the *Ccdc151-lacZ* brain presented in panel A and stained with eosin. Lv, lateral ventricle; d3v, dorsal third ventricle; 3v, third ventricle; Aq, aqueduct of Sylvius. Scale bars: 1 mm (A); 500 μm (B); 50 μm (B, inset).

In order to understand the role of the *Ccdc151* gene in mouse brain development and physiology, we analyzed its expression. Because the *Ccdc151*^–^ allele is a *Ccdc151-lacZ* reporter allele, whole mouse brains were assayed for β-galactosidase enzymatic activity, using X-gal (5-bromo-4-chloro-3-indolyl b-D galactopyranoside) as a substrate in combination with potassium ferri- and ferro-cyanide (X-gal/FeCN protocol) as suggested by protocols developed by the International Mouse Phenotyping Consortium (IMPC) (www.kompphenotype.org) (Skarnes et al., 2011). Positive staining was observed in the brain of heterozygous *Ccdc151^+/−^* animals and not in wild-type controls (Fig. 2A). Visual inspection of the whole-mount stained brain images brought to light a zone of strong staining at the bottom of the brain, in the aperture of the third ventricle, and in the lining of the fourth ventricle separating forebrain, hindbrain and cerebellum (Fig. 2A). The histological analysis demonstrated that *Ccdc151-lacZ* expression was specifically detected in ependymal cells lining the lateral ventricles, third ventricle and aqueduct of the X-gal/FeCN-stained brains (Fig. 2B). Overall, our results highlight that *Ccdc151* is expressed in ependymal cells lining all ventricles reconstituting ventricular compartments of the murine brain.

### 3D X-ray imaging of *Ccdc151-lacZ* reporter gene expression in intact murine brain *in vitro* by microCT

**Fig. 3.**
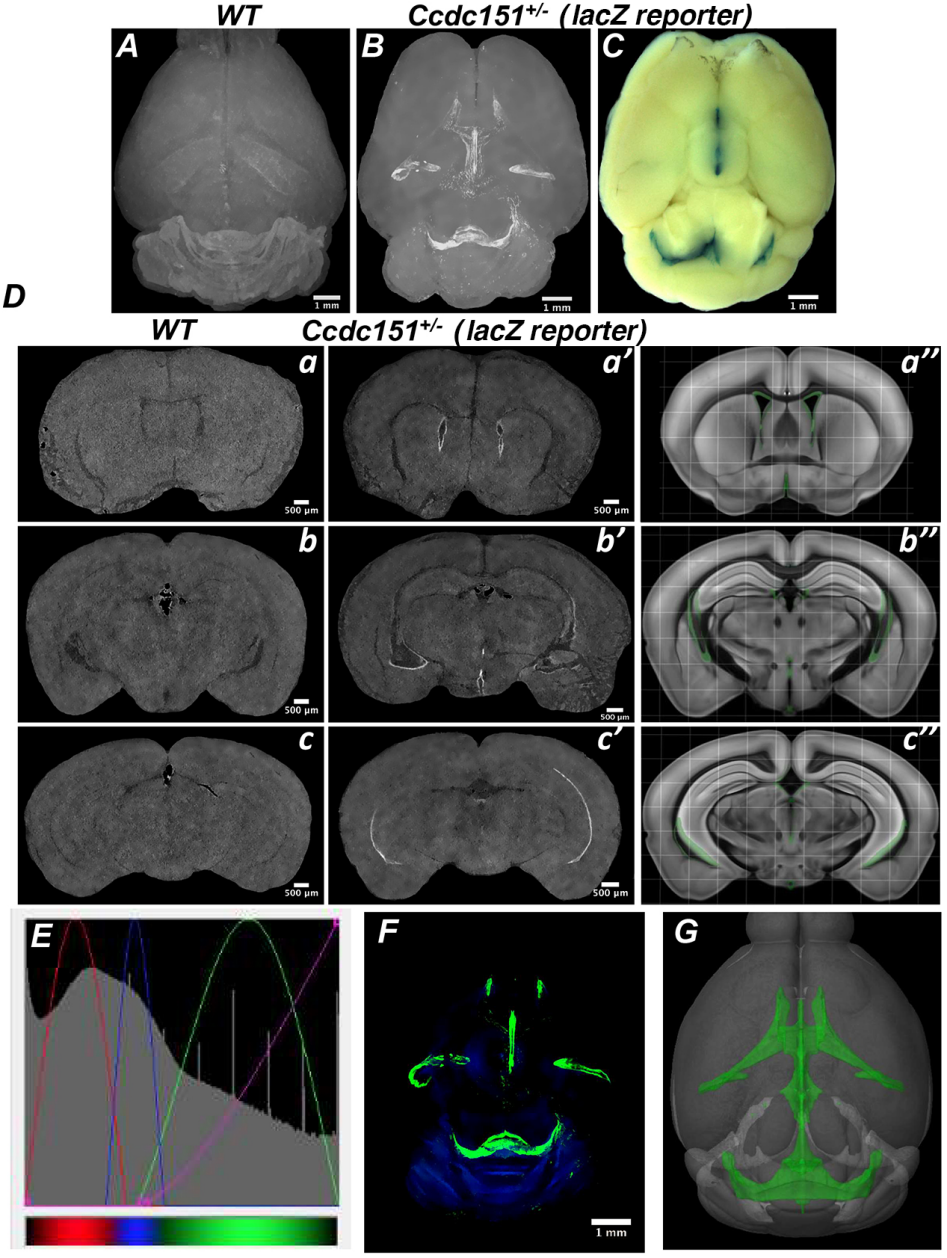
**Three-dimensional microCT imaging of *Ccdc151-lacZ* reporter gene expression in intact mouse brain detects *Ccdc151* expression in the ventricular brain system.** (A) Representative microCT multiple intensity projection (MIP) image of the wild-type (WT) brain stained with X-gal/FeCN and scanned with the resolution of 7.9 µm/voxel. (B) Representative microCT MIP image of the *Ccdc151-lacZ* heterozygous brain stained with X-gal/FeCN and scanned with a 9 µm/voxel resolution. (C) Whole-mount heterozygous brain stained with X-gal/FeCN. (D) Alignment of microCT-derived 2D sections of the WT brain (a-c), microCT-derived sections of the heterozygous *Ccdc151-lacZ* brain (a′-c′) and 2D section derived from a virtually built 3D model obtained using Brain Explorer2 software defining only the ventricular compartment of the mouse brain, marked by green (a″-c″)*.* The regions of increased microCT-detected density (white) in a′-c′ is in remarkable agreement with corresponding virtual 2D coronal sections of the mouse brain, with the ventricular system highlighted in green (a″-c″). (E) Screenshot of the transfer function editor windows of the CTvox analyzer (Bruker software) demonstrates setting of the RGB transfer function curves for building a color volume-rendered 3D model; color coded for the tissue density function: red-blue-green; transparency level defined by the purple line. (F) MicroCT color volume-rendered 3D model of the *Ccdc151-lacZ* brain stained with X-gal/FeCN demonstrates highest densities in the ventricular region of the mouse brain. (G) 3D model of the mouse brain, created using Brain Explorer2 software, defining only the ventricular compartment of the mouse brain (green).

To characterize *Ccdc151* gene expression thoroughly in the whole-mount brain, we took advantage of the power of microcomputed tomography (microCT) analysis. The brain samples from heterozygous *Ccdc151-lacZ* and wild-type animals were stained with X-gal/FeCN, dehydrated, paraffin embedded (as described for histological analysis) and imaged by microCT. Maximum intensity projection (MIP) microCT images revealed regions of high X-ray densities, measured by gray values, only in the *Ccdc151-lacZ* brains (Fig. 3A,B). Regions with large density values were spatially aligned with the β-galactosidase-stained regions observed after whole-mount X-gal/FeCN staining of the *Ccdc151-lacZ-*expressing brains (Fig. 3B,C).

To demonstrate that the observed high densities are due to the β-galactosidase enzymatic activity and originated from the products of its reaction, we compared microCT-derived 2D sections from the *Ccdc151-lacZ* brains with the corresponding sections of wild-type brain (Fig. 3D). Regions with higher densities were specifically observed in the lining of the ventricular system only in *Ccdc151-lacZ* sections but not in the wild-type brains. The regions with positive X-ray densities were in remarkable agreement with the β-galactosidase staining of ependymal cells detected by histological analysis (Fig. 2B).

To further explore the efficacy of X-ray imaging for *lacZ* reporter gene expression analysis, we compared experimentally obtained images to those virtually constructed. We built virtual 2D sections using Allen Mouse Common Coordinate Framework software (https://scalablebrainatlas.incf.org/mouse/ABA12), selecting only the ventricular system zones. The alignment of corresponding virtual and microCT-obtained 2D sections showed a striking colocalization agreement between predicted and our experimental X-ray imaging data (Fig. 3D).

We evaluated the whole microCT datasets using the RGB defined Color Mode (CTvox, Bruker software) and building the corresponding 3D volume distribution of *Ccdc151-lacZ* expression (Fig. 3E,F, Movie 1). The model displayed that the high X-ray dense regions are restricted to ventricular system and match considerably with the corresponding ventricular regions in the digital 3D Allen Mouse Brain Atlas (https://mouse.brain-map.org/; Fig. 3G, Movie 2). Therefore, using a novel custom microCT imaging protocol, which is based on the detection of β-galactosidase*/*X-gal reaction products in intact murine brain *in vitro*, we further demonstrated that *Ccdc151* is expressed in the ventricular system of the murine brain.

### *Ccdc151*-knockout animals develop communicating hydrocephalus

Above, we showed that *Ccdc151*-null animals developed a dome-shaped head, indicative of hydrocephalus, at the second postnatal week. We also demonstrated expression of *Ccdc151* in ependymal cells of the ventricular system of the brain, suggesting a key role of *Ccdc151* for normal ventricular system functions. This observation is in agreement with previously published data showing that loss of function due to point mutations in the mouse *Ccdc151* gene (*Snowball* mutants) leads to immotile and dyskinetic cilia, including cilia on ependymal cells of murine brain (Hjeij et al., 2014).

**Fig. 4.**
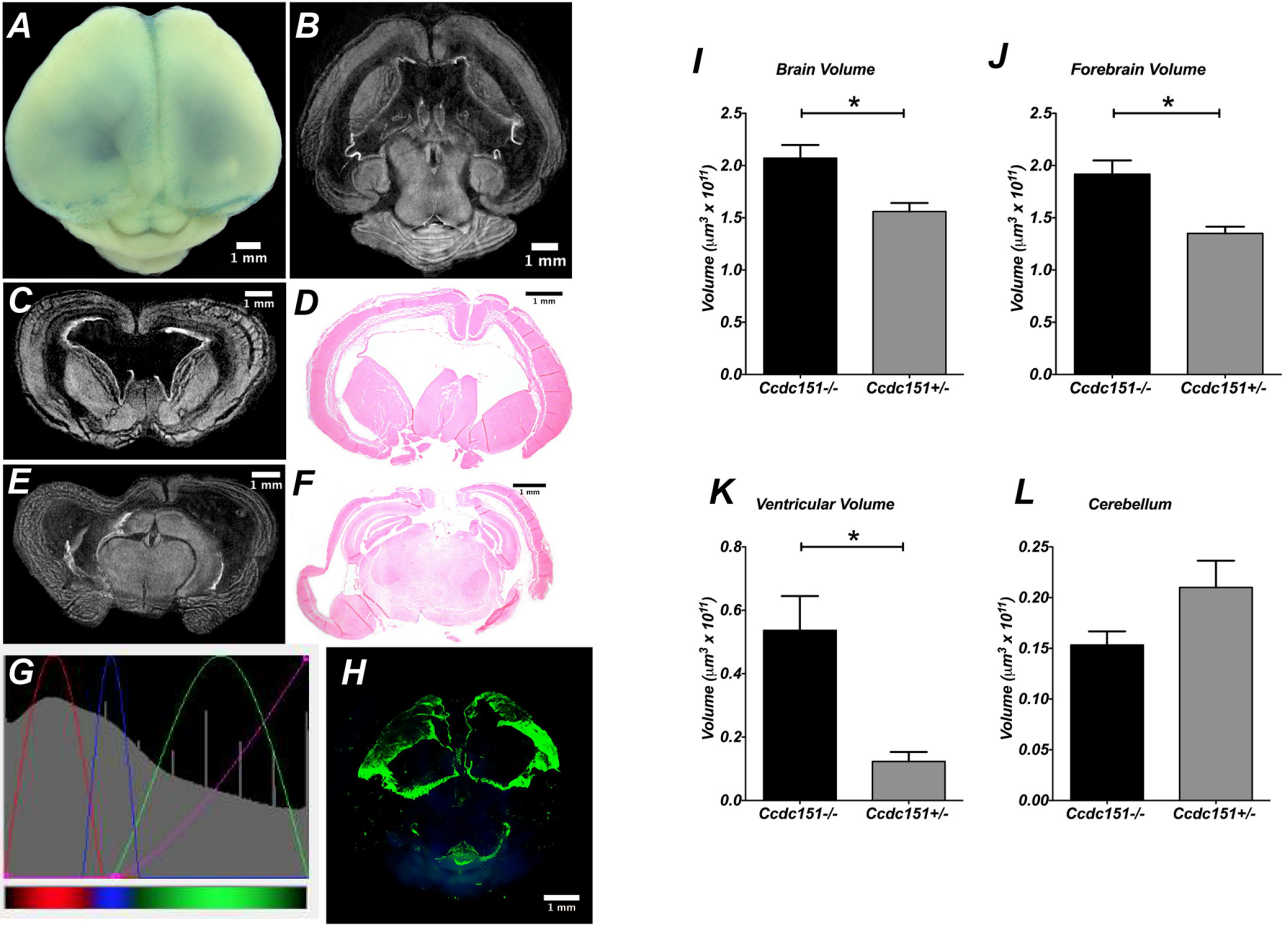
***Ccdc151*-knockout animals develop postnatal hydrocephalus.** (A) Dorsal view of mouse brain from homozygous *Ccdc151^−/−^* animals stained with the X-gal/FeCN protocol. (B) MicroCT-derived transversal sections of the *Ccdc151^−/−^* brain demonstrate enlargement of the lateral ventricles. (C-F) Coronal sections of the *Ccdc151^−/−^* knockout brain; microCT-derived coronal sections of the forebrain (C,E) and corresponding histological sections (D,F) demonstrate dilation of lateral ventricles and cortex thinning. (G) Screenshot of the transfer function editor windows of the CTvox analyzer (Bruker software). Settings of the RGB transfer function curves for building a color volume-rendered 3D model, color coded for the tissue density increases: red-blue-green; transparency level defined by the purple line. (H) MicroCT color volume-rendered 3D model of the *Ccdc151^−/−^* knockout brains stained with X-gal/FeCN demonstrate highest densities in the ventricular region of the mouse brain. Ependymal cell integrity is interrupted in the dorsal part of the lateral ventricles. (I-L) Quantification of the pathological hydrocephalus of the murine brain of *Ccdc151^+/−^* (*N*=3) and *Ccdc151^−^*^/−^ (*N*=3) at P12. (I) Measurements of the total brain volume; mean+s.e.m.; Mann–Whitney one-tailed *t*-test, *P=0.05. (J) Forebrain volume; mean+s.e.m.; Mann–Whitney one-tailed *t*-test, *P=0.05. (K) Ventricular volume; mean+s.e.m.; Mann–Whitney one-tailed *t*-test, *P=0.05. (L) Cerebellar volume; mean+s.e.m.; Mann–Whitney one-tailed *t*-test, *P*=0.09 (non-significant).

Here, using microCT imaging and histological analysis of *Ccdc151^−/−^* brains, we observed that homozygous-null animals develop severe hydrocephalus with complete penetrance of this phenotype. The brain of *Ccdc151^−/−^* animals was characterized by forebrain enlargement, due to massive expansion of lateral ventricles, cortex thinning and cerebellar compression (Fig. 4A-F). Often, hemorrhages within the cortex of null animals were observed (data not shown). The above listed features were not detected in *Ccdc151^+/−^* and wild-type brains of littermates (Fig. 3). We used microCT 3D imaging for anatomic and quantitative characterization of the hydrocephalic features in the brain of *Ccdc151*-null animals. Because we previously found that *Ccdc151* is expressed in ependymal cells, we took advantage of this observation to analyze homozygous-null *Ccdc151-lacZ* animals in order to mark ependymal cells for examination of the ventricular system. With this aim, we built an RGB defined Color Mode 3D model (Fig. 4G,H, Movie 3). Close inspection of the movies confirmed an enlargement of the lateral ventricles. In addition, the dorsal part of lateral ventricles were deprived of the ependymal cell layer. It is still unclear whether the interruption of ependymal-cell-lining integrity is a consequence of the extreme ventriculomegaly observed in *Ccdc151*-null animals or whether the initial denudation occurs prior to massive ventricular enlargement. Future microCT volume imaging inspections of *Ccdc151-lacZ* ependymal cell integrity during immediate postnatal brain development and hydrocephalic progression in *Ccdc151*-null animals will allow us to address this issue.

Volumetric analysis of *Ccdc151^−/−^* brains was performed by microCT imaging. A significant increase in total brain and forebrain volumes as well as ventricular volume were found in the *Ccdc151^−/−^* animals, when compared to *Ccdc151^+/−^* littermates (Fig. 4I-K). However, analysis of our data did not reveal statistically significant differences between the two genotypes when cerebellar volumes were examined, suggesting that the main phenotypic consequences of the *Ccdc151* gene knockout point toward modifications of the forebrain structures in *Ccdc151^−/−^* animals (Fig. 4L).

**Fig. 5.**
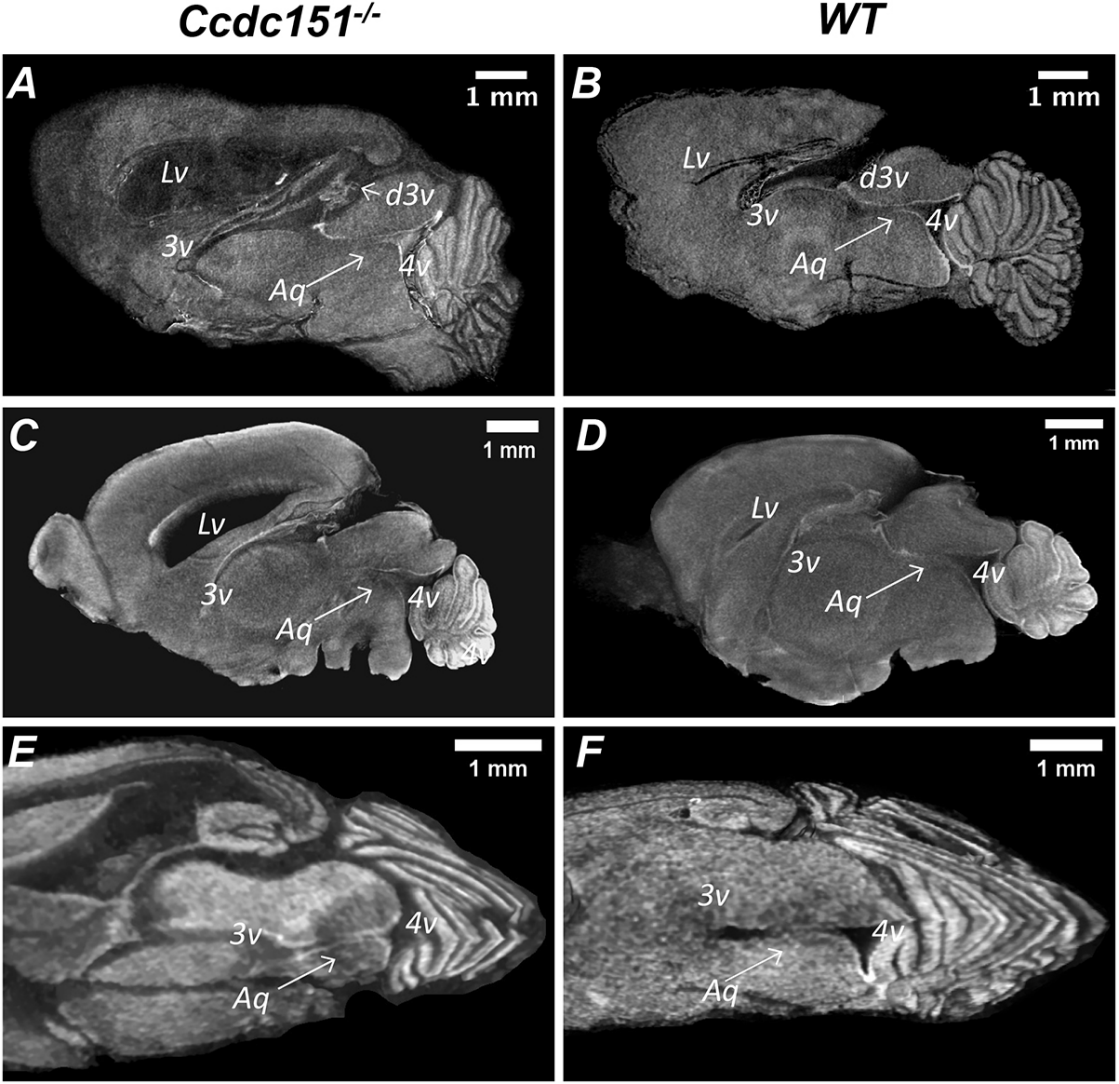
***Ccdc151-*knockout animals develop communicating hydrocephalus.** (A,B) MicroCT analysis of paraffin-embedded whole-mount brains imaged at a resolution of 7.9 µm voxel size. Virtual microCT-derived sagittal sections from the brain of *Ccdc151^−/−^* (A) and wild-type (WT) animals (B) demonstrate continuity of the aqueduct of Sylvius. (C,D) MicroCT imaging analysis of the brains treated with Lugol's solution as a contrasting agent. Brains were imaged at 6.9 µm voxel size resolution. Virtual microCT-derived sagittal sections of the *Ccdc151^−/−^* (C) and WT (D) brains. (E,F) Virtual dissection of the brains by placing transversal and sagittal planes across the paraffin-embedded brains from *Ccdc151^−/−^* (E) and WT (F) animals. Lv, lateral ventricle; d3v, dorsal third ventricle; 3v, third ventricle; 4v, fourth ventricle; Aq, aqueduct of Sylvius.

We next examined whether hydrocephalus could be caused by anatomical abnormalities of the cerebral aqueduct of Sylvius. Virtual dissection of the microCT-derived brain volumes allows for examination of collected images at different frames and angles. We first compared the aqueductal path between *Ccdc151^−/−^* and wild-type brains embedded in paraffin. Well-defined aqueductal structures were observed in both *Ccdc151*-knockout and wild-type animals (Fig. 5A,B). To examine whether this assessment is true independently of the experimental protocol applied for sample preparation, we used a different method to prepare the samples for microCT imaging. Whole murine brains dissected from the scalp were incubated in Lugol solution, a contrasting agent routinely used for microCT imaging of soft tissues. The Sylvius aqueductal structure was clearly visible and looked normally developed on X-ray images obtained from the mouse brains treated with Lugol solution, suggesting that this observation did not depend upon the method by which samples were treated prior to X-ray imaging (Fig. 5C,D). Some deviation from the perfect anatomical aqueductal structure observed in *Ccdc151*-null animals could be explained by general growth malformation of the hydrocephalic brains.

**Fig. 6.**
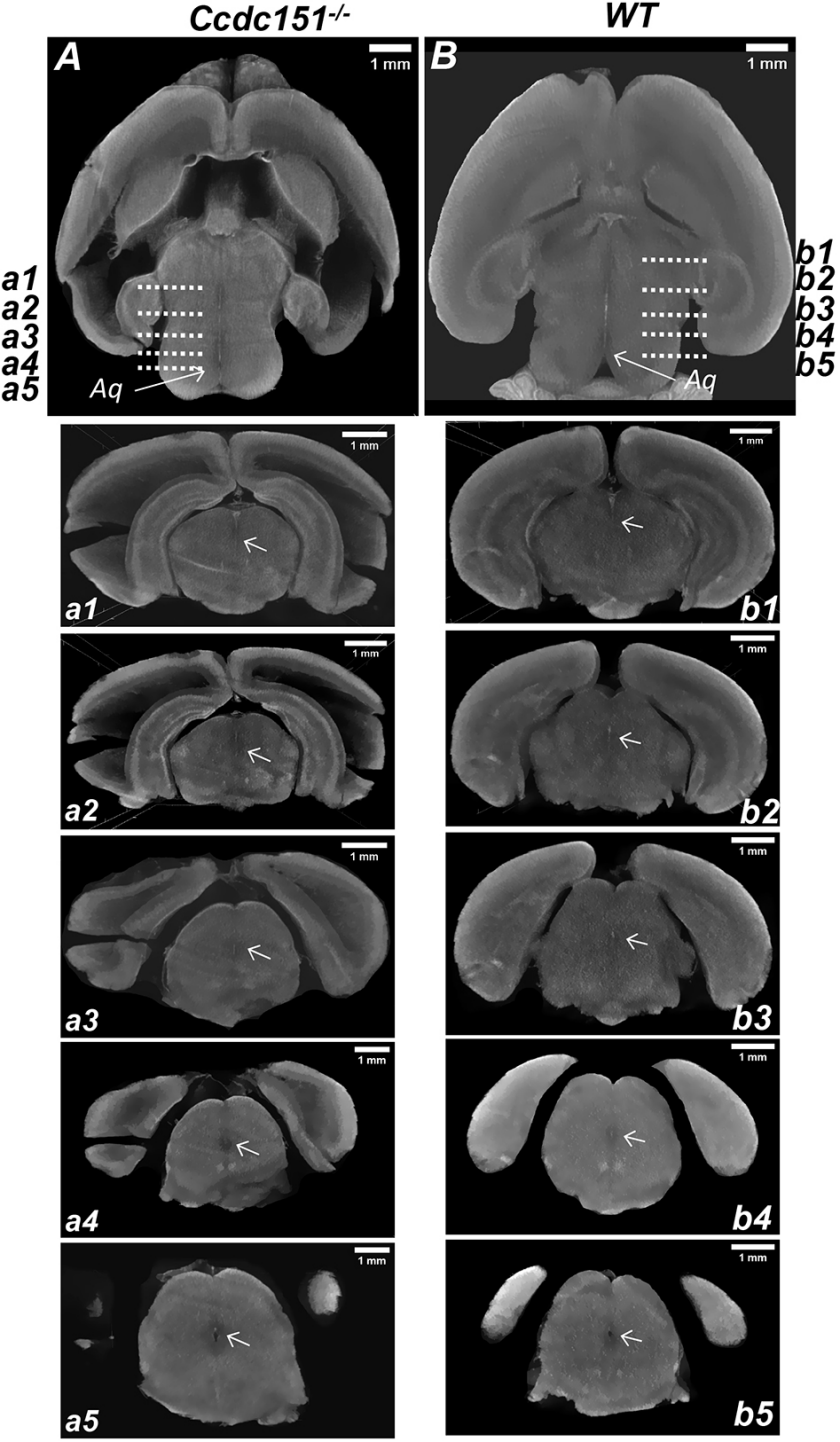
**MicroCT analysis of** **continuity of the** **aqueduct** **of Sylvius.** (A,B) Virtual microCT-derived transversal sections from the brain treated with the Lugol contrasting agent and imaged with a resolution of 6.9 µm voxel size. The continuity of the aqueduct in the brain of *Ccdc151^−/−^* (A) and wild-type (WT; B) animals is indicated by an arrow. Sequence of sagittal sections demonstrating aqueduct continuity from the brain of *Ccdc151^−/−^* (a1-a5) and WT (b1-b5) animals. Arrows indicate aqueduct.

To evaluate the continuity of aqueductal path, we further virtually dissected microCT-reconstructed brain volumes, placing two perpendicular planes (sagittal and transversal) through the line of the aqueductal path (Fig. 5E,F). This analysis showed a continuous aqueductal passage without any visible obstruction, suggesting that the hydrocephalus does not occur because an anatomical discontinuity in Sylvius aqueduct. Continuity of the aqueductal path was also confirmed in samples treated with Lugol solution (Fig. 6). Therefore, our results indicate that the loss of *Ccdc151* gene function causes communicating hydrocephalus in mice.

### *Ccdc151*-deficient mice show defects in left-right body asymmetry

**Fig. 7.**
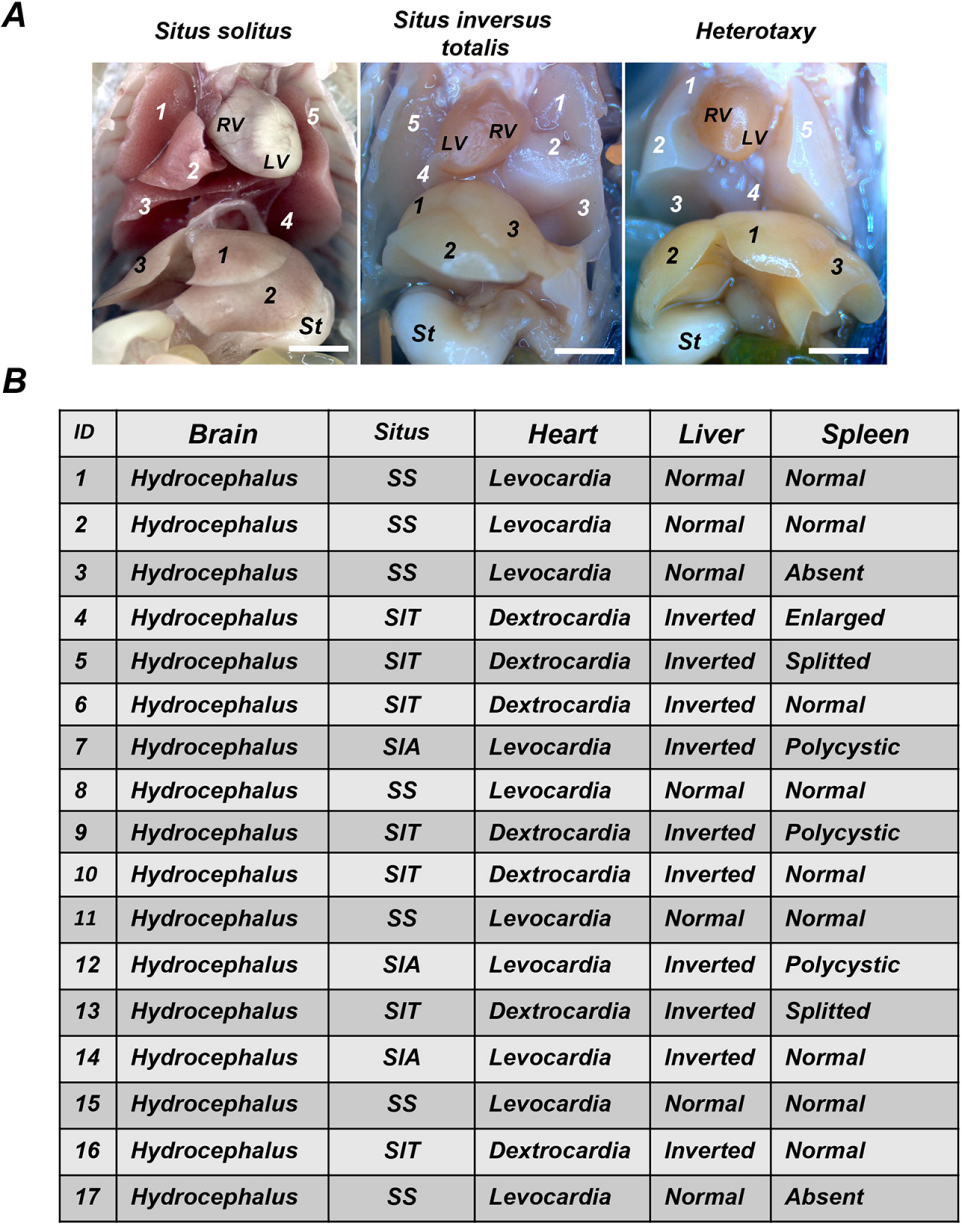
**Targeted deletion of *Ccdc151* in mice leads to left-right asymmetry defect, a trait of the PCD disease.** (A) *Ccdc151*-knockout animals presented with situs solitus (SS), situs inversus totalis (SIT) and situs inversus abdominalis (SIA). RV, right ventricle; LV, left ventricle; St, stomach; 1-5 (white numbers), numeration of lung lobes; 1-3 (black numbers), numeration of liver lobes. (B) Summary of the phenotypes observed in *Ccdc151*-knockout animals.

Defects in laterality are a common feature of PCD disease. We observed that *Ccdc151^−/−^* animals can have either normal position of the organs, situs solitus (SS) or a complete mirror-image inversion of organs from the left to right site (SIT) (Fig. 7A). We analyzed 17 *Ccdc151^−/−^* animals and noticed similar frequency between SS and SIT (7 animals, respectively), with only three animals having SIA. A total of 41% of *Ccdc151^−/−^* animals presented with normal left-right asymmetry and 69% had situs abnormalities (Fig. 7B). Morphological examination of other organs in *Ccdc151*-null animals evidenced spleen abnormalities such as being polycystic, split at the distal end or missing (asplenia) (Fig. 7B). Asplenia and polysplenia are often detected in patients with situs ambiguous (Lin et al., 2014). Situs ambiguous generally refers to randomization of organ position in the body, including right (two right-sides) or left (two left-sides) isomerisms. Interestingly, here, asplenia was observed in animals with SS, suggesting that correct cilia functions might be important for spleen organogenesis independently of left-right body-axis establishment. To confirm this observation, further systematic analysis of *Ccdc151*-null animals is required.

### *Ccdc151* is required for normal spermatogenesis

**Fig. 8.**
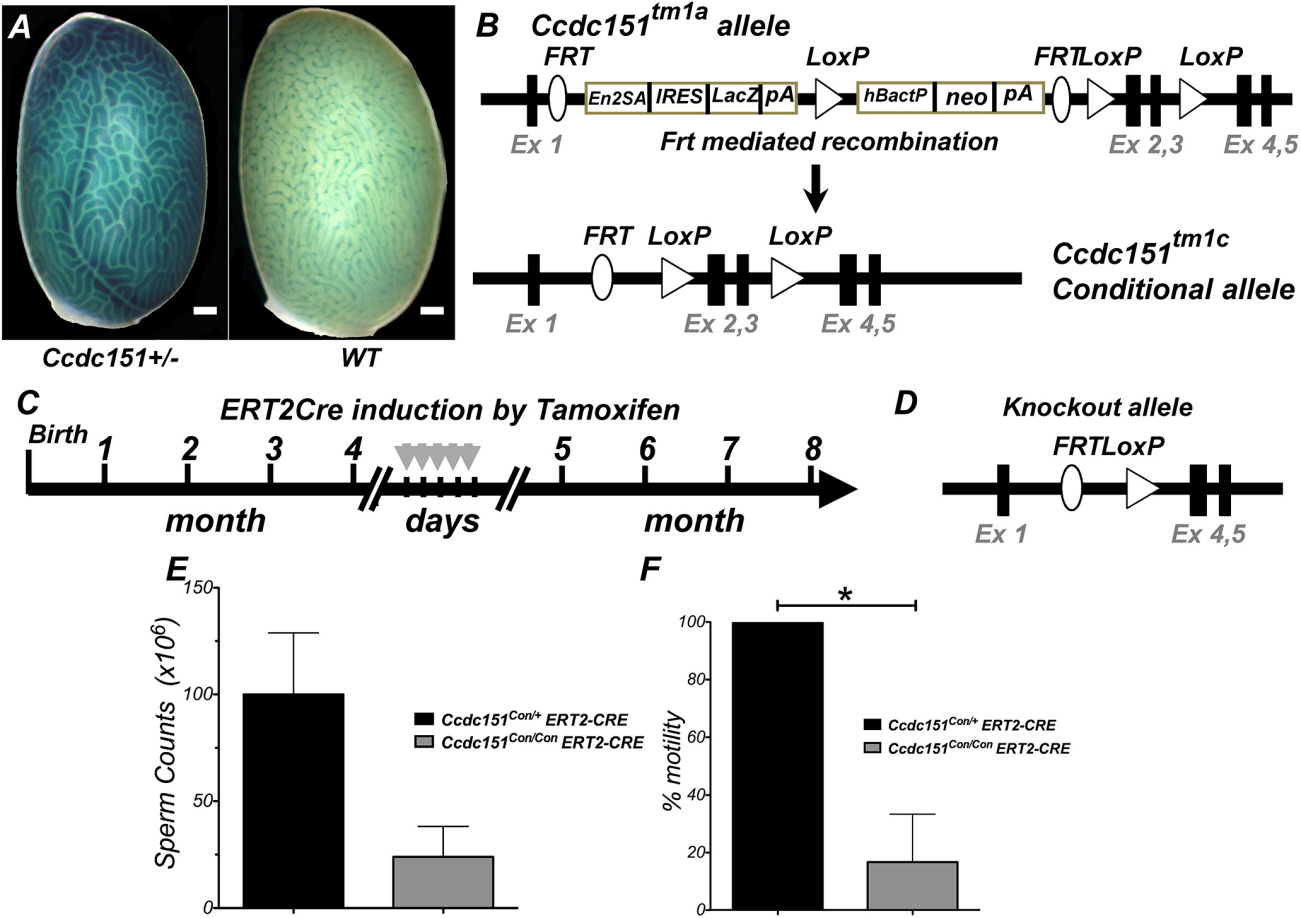
**The** ***Ccdc151* gene is expressed in testes and** **its** **targeted deletion leads to defective** **spermatogenesis.** (A) Whole-mount staining of testes from *Ccdc151-lacZ* animals and wild-type (WT) control using the X-gal/FeCN protocol, demonstrating expression of *Ccdc151* in testicular seminiferous tubules of the testis. Scale bars: 500 μm. (B) Schematic representation of the conversion of the *Ccdc151^t^^m1a^* allele into the conditional allele *Ccdc151^t^^m1c^* upon FLP-dependent recombination. Ex, exon; *En2ASA*, splice acceptor site; *IRES*, internal ribosomal entry site; *LacZ*, bacterial β-galactosidase reporter gene; *pA*, poly A; *hBactP*, human β-actin promoter; *neo*, neomycin resistance gene; *FRT*, FLP recombination sites; *LoxP*, Cre recombination sites. (C) Schematic representation of *ROSA26ERT2-Cre* recombinase induction by tamoxifen injections. (D) Schematic representation of the *Ccdc151* knockout allele, after induction of Cre recombination. (E,F) Characterization of spermatogenesis after induction by tamoxifen of Cre recombinase in *Ccdc151^Con/Con^ ERT2-Cre* (*N*=3) and *Ccdc151^Con/+^ ERT2Cre* (*N*=3) animals: sperm count, mean+s.e.m.; Mann–Whitney *t*-test, *P*=0.06 (E) and % of sperm motility, mean+s.e.m.; Mann–Whitney *t*-test, *P=0.05 (F).

PCD syndrome is often associated with male infertility in humans and mice. To address the role of *Ccdc151* in spermatogenesis, we first analyzed whether *Ccdc151* is expressed in the testes of adult animals. Taking advantage of the *Ccdc151-lacZ* reporter allele, we stained whole testes using the X-gal/FeCN protocol and observed very strong expression in testicular seminiferous tubules (Fig. 8A). Most *Ccdc151*-null animals died before sexual maturity, so hampering a systematic fertility analysis. In our study, only three males survived post-weaning and progressed to sexual maturation age. In those surviving *Ccdc151*-knockout animals, we observed that the epididymis and vas deferens were correctly developed; however, a complete absence of spermatozoa was found (Movies 4, 5). Observed azoospermia in *Ccdc151*-null animals suggested the occurrence of some defect in sperm maturation and/or sperm retention in the efferent ductules of male testicles.

To further examine the role of *Ccdc151* for fertility in adults, we produced animals with an inducible conditional allele for this gene. To generate a conditional allele, *Ccdc151^tm1a(EUCOMM)Hmgu^*/Cnrm animals were fist bred with a FLP-recombinase-expressing strain (MGI:2429412). The resulting conditional *Ccdc151^tm1c^* (*Ccdc151^Con^*) allele has exons 2 and 3 of the *Ccdc151* gene floxed by the *loxP* sites (Fig. 8B). Homozygous *Ccdc151^Con/Con^* animals were viable and apparently normal. To delete the *Ccdc151* gene in adult animals, we mated *Ccdc151^Con/Con^* with *ROSA26* transgenic animals expressing Cre recombinase inducible by tamoxifen (*ROSA26ERT2-Cre*; MGI:3764519). This strategy allowed deletion of the *Ccdc151* gene in adult animals by injection of tamoxifen (Fig. 8C,D). After Cre recombinase induction, both sperm counts and sperm motility were greatly reduced in the *Ccdc151^Con/Con^ ERT2Cre* animals but not in the heterozygous *Ccdc151^Con/+^ ERT2Cre* littermates (Fig. 8E,F). While it is predictable that the sperm motility would be affected because of the possible role of *Ccdc151* in flagella motility, the dramatic decrease in the sperm counts in adult animals requires further study.

## DISCUSSION

In this work we describe how the loss of *Ccdc151* in mice leads to the development of a severe postnatal, rapidly progressing hydrocephalus and perinatal lethality. This mouse model is the first example of a hydrocephalus phenotype caused by loss of function of the *Ccdc151* gene. Such a role of the *Ccdc151* gene in hydrocephalus development is further supported by the analysis of its expression. Using animals expressing the *Ccdc151-lacZ* reporter allele we demonstrated that *Ccdc151* gene expression is localized in ependymal cells lining the ventricular brain system. While we did not characterized cilia in the *Ccdc151^−/−^* animals, another group that used the *Ccdc151^Snbl^* animal model, carrying a loss-of-function point mutation in the *Ccdc151* gene, observed defective ciliary motility. The ependymal cilia lining the brain ventricles of *Ccdc151^Snbl^* mutants were largely immotile, with occasional patches exhibiting slow and stiff ciliary motion (Hjeij et al., 2014). In spite of observed ciliary defects, the hydrocephalus was not reported in *Ccdc151^Snbl^* animals. The differences in hydrocephalus phenotype between the two *Ccdc151* loss-of-function animal models could be attributed to: (1) the genetic difference in nature of the two mutant *Ccdc151* alleles (point mutation versus exons deletion), or (2) the genetic background of the animals in which phenotypic analysis was conducted. *Ccdc151^Snbl^* animals were produced via ENU mutagenesis in the C57BL/6J strain, and were maintained and analyzed on this background (MGI: 5445347), while we produced and maintained *Ccdc151^−/−^* animals on a pure C57BL/6N genetic background. This observation suggests that genetic modifiers of a hydrocephalic phenotype might influence the susceptibility to hydrocephalus in *Ccdc151* loss-of-function murine models. The role played by the genetic background for manifestation of hydrocephalus was previously demonstrated in mouse models of PCD lacking ciliary proteins CFAP221, CFAP54 and SPEF2, respectively. In all these three loss-of-function mouse models, the severity of hydrocephalus was increased when the animals were maintained on a C57BL/6J (B6) background, compared to the same models on 129S6/SvEvTac (129) or a mixed (B6×129)F1 background (Horani et al., 2016; Flinn et al., 2014; McKenzie et al., 2018). Interestingly, CSF flow in these mice was perturbed on both backgrounds, indicating that abnormal cilia-driven flow is not the only factor underlying the hydrocephalus phenotype. Indeed, a comparative gene expression microarray analysis of a mice lacking *CFAP221* on the B6 and 129 backgrounds reveals differences for a number of genes implicated in cellular and biochemical processes essential for proper brain development as well as for formation and function of both motile and sensory cilia (McKenzie et al., 2018). Importantly, because the C57BL/6N strain arose from C57BL/6J (Simon et al., 2013), these two substrains are much more genetically closer than B6 and 129, implying that a comparative dissection of the genetic modifier(s) of hydrocephalus development due to loss of *Ccdc151* function could be substantially facilitated by fewer genetic variations between C57BL/6N and C57BL/6J substrains.

Hydrocephalus is often associated with structural abnormalities acquired during brain development. Mutations in genes responsible for normal neuronal progenitor cell division, differentiation, maturation or migration, such as *TRIM71*, *SMARCC1*, *PTCH1*, *SHH* and *L1CAM*, or in genes encoding cell adhesion proteins important for the ependymal cell maturation and ependymal layer integrity, such as *MPDZ*, *YAP* and *JAM-C*, leads to hydrocephalus in humans and mice (Furey et al., 2018; Feldner et al., 2017; Tonosaki et al., 2014; Park et al., 2016). It is important to note that all of the examples documented above lead to non-communicating hydrocephalus, which occurs due to anatomical and morphological defects, such as severe stenosis or complete occlusion of the aqueduct of Sylvius, and is caused by abnormally developed or migrating ependymal cells forming anatomical structures that physically block normal CSF circulation.

Structural defects in the ciliary axoneme of ependymal cells that cause inadequate cilia motility, CSF flow slowdown or changes in its directionality are other common causes of hydrocephalus. This phenotype is often accompanied by other PCD-associated traits in mice. In Table S1 we list some of the available literature describing the mouse models with knockout of axonemal genes with PCD phenotypes. Interestingly, when the anatomical features of hydrocephalus in these animals were analyzed, all but one exhibited the communicating type of hydrocephalus. The only exception was the hydrocephalus observed in mice with a null mutation in the *Dnah5* gene, which encodes the axonemal dynein heavy chain (Ibañez-Tallon et al., 2004). This murine model was the first of many ascertaining a relationship between defective cilia motility and hydrocephalus. *Dnah5*-knockout animals developed a very early severe and fast-progressing hydrocephalus with early postnatal lethality. *Dnah5*-null and *Ccdc151*-knockout animals both show very similar phenotypic traits, such as hydrocephalus, early postnatal lethality and defect in left-right body axis specification. These findings are in accordance with the fact that deletion of the *Ccdc151* gene abrogates *Dnah5* binding to the ciliary axonemes, consistent with a defect of ODA assembly in axonemes of *Ccdc151^Snbl^* cilia and defective ciliary motility (Hjeij et al., 2014). Morphological characterization of the *Dnah5*-null brains by histological analysis of the paraffin-embedded brain demonstrated discontinuity of the aqueduct of Sylvius by blockage between proximal and distal parts of the aqueductal path. Instead, in paraffin-embedded and Lugol-treated whole-mount brains of *Ccdc151*-null animals, we found aqueductal integrity as ascertained by microCT imaging. Our results indicate that the aqueduct is normally formed and visible path obstruction does not occur, suggesting that *Ccdc151*-knockout animals develop communicating hydrocephalus. This is an important point because it was previously hypothesized that ependymal CSF propulsion into the aqueduct is required to prevent aqueductal stenosis during early postnatal development (Ibañez-Tallon et al., 2004).

The observed discrepancy between the two described murine models could be due to the different methodologies used for the morphological characterization of the hydrocephalic brains. In our studies we applied 3D analysis by microCT of the whole mouse brain, which allows for a thorough examination of the intact internal structures of the brain, while histological 2D analysis, which was applied for analysis of the *Dnah5*-knockout brain, is more invasive because the required histological sectioning often makes small structures, like the aqueduct, prone to collapsing. This hypothetical explanation could be easily verified by analyzing the brain of *Dnah5*-knockout animals by microCT in the same way we did.

In humans, PCD rarely links with hydrocephalus. In addition, axonemal genes were not found among the genes causing congenital hydrocephalus in patients (Tully and Dobyns, 2014). Therefore, to what extent defective ependymal ciliary motility contributes to the development of congenital hydrocephalus is still a matter of debate (Afzelius, 2004). The presence of patients who have been diagnosed with hydrocephalus (or with enlarged ventricles only) and with immotile, dysmotile or lacking cilia suggests the existence of a genetic link between these two phenotypic traits (Jabourian et al., 1986; De Santi et al., 1990; Picco et al., 1993; Al-Shroof et al., 2001; Wessels et al., 2003). Yet, so far the only gene found to be mutated in patients affected by PCD with mild ventricular enlargement is the *DNAH5* gene (Ibañez-Tallon et al., 2004). Therefore, species-specific differences might explain why, in humans, PCD does not occur with severe hydrocephalus, despite this often being observed in mice. Another conceivable explanation is based on noticing that the minimum width of the mouse foramen of Monro (<10 nm) is 100,000-fold smaller than in humans (>1 mm), so facilitating the circulation of CSF in human brain (Lang, 1992). Moreover, the erected position adopted by humans during evolution might help CSF to flow due to gravity compensating for the less strict requirement of normal ciliary motility.

*Ccdc151* deficiency causes PCD with randomization of left-right body asymmetry in human patients and in *Ccdc151^Snbl^* animals (Hjeij et al., 2014). Here, we demonstrated that the targeted deletion of the *Ccdc151* gene leads to defects in left-right body asymmetry such as SIT and SIA. We observed a similar frequency for SIT and SS in our animals (41% each), while only 18% had SIA. Our data are in agreement with anatomical studies of PCD patients, which showed that about half had laterality defects and around 12% manifested complex isomerism (Mirra et al., 2017). It is interesting to note that, although only relatively few mutated genes important for ciliary motility lead to hydrocephalus in murine models, the laterality defects are more frequent (Table S1), suggesting that correct motile cilia functioning during left-right asymmetry acquisition is tightly regulated and these events are much more sensitive to small structural and functional irregularities, which are translated in situs abnormalities.

Male infertility is another distinctive and quite common pathology in PCD patients. It is characterized by an ample degree of sperm abnormalities, ranging from reduced sperm motility to complete azoospermia (Munro et al., 1994). For example, mutation in *DNAH1*, encoding a component of IDA3, has been linked to azoospermia in patients (Ben Khelifa et al., 2014). This is not surprising because spermatozoa flagella and motile cilia share the same highly conserved 9+2 microtubules doublet axoneme structure (Afzelius, 2004). In addition, motile cilia are also present in the cells of the male reproductive tract. The role of motile cilia in efferent ductules is to create a condition for the effective transport of immotile sperm from the rete testis to epididymis. Indeed, the loss of efferent duct motile cilia causes sperm aggregation and agglutination, as well as luminal obstruction, which, in turn, induce back-pressure atrophy of the testis and ultimately male infertility (Yuan et al., 2019).

The fertility phenotype in mouse models of PCD is often obscured because of the severe congenital abnormalities in other physiological systems and death before sexual maturation. Most *Ccdc151*-null animals die before testis maturation, precluding fertility assessment in males. The strategy to conditionally induce *Ccdc151* knockout in adult mice allows this problem to be overcome, providing a valuable model for analysis of the factors causing infertility in *Ccdc151*-null males. Indeed, using the conditional approach, we found that a dramatic drop both in spermatozoa counts and motility occurs at 3 months post-ablation of the *Ccdc151* gene. Importantly, a high percentage of produced spermatozoa are characterized by impaired or complete absence of motility. This observation suggests that *Ccdc151* is required for flagella movement and sperm motility. The reason for the high drop in sperm number after induction of the *Ccdc151* gene knockout is still unclear but we hypothesize that it could occur because of the loss of cilia beat in compartments of spermatozoa maturation that is required for normal spermatogenesis. In summary, we identified *Ccdc151* as a gene critical for sperm maturation and motility.

To accomplish our work, we extensively exploited X-ray microCT 3D imaging analysis for the morphological and volumetric analysis of hydrocephalic *Ccdc151^−/−^* brains. This method greatly facilitates the analysis of hydrocephalic brains. Hydrocephalic brains filled with CSF liquid are prone to mechanical damage during histological sectioning of the samples. We found that dehydrated and paraffin-embedded brains allow for visualization and morphometric analysis of total brain and ventricular volume, with manipulation of the sample and other experimental efforts being kept at a minimum. This highly conservative method is extremely convenient and could be applied for phenotypic characterization of other hydrocephalic mouse models, since quantification of small variations of the ventricular volume can be easily achieved in large cohorts of animals, which are necessary for the statistical validation of the experimental data.

In this work, we also introduce a novel microCT method for gene expression detection and visualization of genes carrying the *lacZ* reporter gene in whole-mount mouse brains *ex vivo*. This method is based on generating a molecular signal within the mouse brain through the reaction catalyzed by the bacterial enzyme β-galactosidase, which is widely used as a reporter gene in many mouse genetic experiments. Comparing histological 2D data and those obtained from microCT analysis of *Ccdc151-lacZ* expression, we observed an excellent agreement between the two mapped gene expression distributions, demonstrating a specific expression of the *Ccdc151* gene in ependymal cells lining the ventricular system. Therefore, we concluded that specific densities obtained by an *in situ* β-galactosidase reaction, detectable by X-ray imaging, are direct and genuine signals of the corresponding *lacZ* gene reporter expression. In addition, by labeling the ependymal cells with *lacZ* reporter and using volume microCT imaging, we provide a new tool to study hydrocephalus development. This procedure allows focus on the development of the ependymal layer and validation of the changes in its integrity during the pathogenesis of hydrocephalus.

In conclusion, the work presented in this paper demonstrates that animals with *Ccdc151* knockout are an important model of PCD. Furthermore, a novel microCT imaging approach for 3D morphometric and gene expression analysis to study hydrocephalus development in mice is introduced in this work. Overall, this *Ccdc151* loss-of-function murine model has proven to be a valuable model to study specific pathological phenotypic traits associated with ciliary dysfunction during development and in adult animals.

## MATERIALS AND METHODS

### Ethics statement

All animals used were handled in accordance with the experimental protocols and animal care procedures reviewed and approved by the Ethical and Scientific Commission of Veterinary Health and Welfare Department of the Italian Ministry of Health (protocol approval reference: N:0000688 03/21/2008). The ethical and safety rules and guidelines for the use of animals in biomedical research provided by the Italian laws and regulations, in application of the relevant European Union's directives (n. 86/609/EEC and 2010/63/EU), were adhered to.

### Animals

Mice analyzed in this study were produced by CNR Monterotondo Mouse Production Unit and maintained on a C57BL/6N background by the European Mouse Mutant Archive (EMMA) facility in Monterotondo, Rome, Italy. Animals were maintained in a temperature-controlled room at 21±2°C, on a 12-h light-dark cycle (lights on at 7 a.m. and off at 7 p.m.). After weaning, mice were housed by litter of the same sex, 3-5 per cage, with food and water available *ad libitum* in a specific pathogen-free facility. *Ccdc151^tm1b^/+* mice were produced by IKMC at Monterotondo, Italy. *Ccdc151^tm1a(EUCOMM)Wtsi^* ES cells were injected into blastocysts and chimeras were produced. Obtained chimeras transmitted the targeted allele into progenies. The *neo* selection cassette flanked by *loxP* sites was excised upon breeding with *ROSA26Cre* (MGI: 3764519) animals. By this breeding strategy, heterozygous animals carrying the *Ccdc151^tm1b^* allele were generated. Genotyping of the *Ccdc151^tm1b^* allele was performed with the following primers (5′-3′): *Ccdc151* F: AGAGCCCTGGATCTTAACTGCTGA, *Ccdc151* R: TCCAAGTCATGCAGAGCTGGGATT and *Frt-Rev*: CCTTCCTCCTACATAGTTGGCAGT; the wild-type allele produces a PCR product of 307 bp, while the *Ccdc151^tm1b^* allele is 270 bp in size.

### *Ccdc151* transcript analysis in brain

RNA was extracted from mouse brain using Trizol reagent according to the manufacturer's protocol (Invitrogen, cat. # 15596-018). In total, 500 ng were used to prepare cDNA (Invitrogen, 18080093). The PCR reactions using cDNA as template were performed with the following primers (5′-3′): pair 1: F- CTCCATACGCTCTGTCTGAA and R- TGCGCAACCTGGAGAATCGGC, and pair 2: F- TGATACGGTATTGGCAGGAA and R- TAAGATCTGCGCAACCTATT; GAPDH F- ATGTGTCCGTCGTGGATCTGAC and R- AGACAACCTGGTCCTCAGTGTAG.

### Whole-mount *lacZ* gene reporter assay with X-gal (X-gal/FeCN protocol)

The animals were perfused as described above; the organs were dissected and fixed in 4% PFA on ice for 30 min and then were incubated for 30 min in wash buffer (2 mM MgCl_2_, 0.01% sodium deoxycholate, 0.02% of Nonidet-P40, 0.1 M sodium phosphate buffer, pH 7.3) on ice. Whole-mount organs were stained in X-gal staining solution containing 1 mg/ml of X-gal stock solution [25 mg/ml X-gal (Sigma-Aldrich, B4252) in dimethylformamide], 0.2% of potassium ferrocyanide (Sigma-Aldrich, P-9387) and 0.16% potassium ferricyanide (Sigma-Aldrich, P-8131) in wash buffer for about 48 h at 37°C. The organs were washed in wash buffer and additionally fixed overnight with 4% PFA in the cold room and embedded in paraffin as described (Marazziti et al., 2013). The embedded organs were then sectioned for histological analysis or inspected by microCT imaging. When whole-mount X-gal staining was not performed immediately after perfusion, organs were fixed with 4% PFA overnight in the cold room and embedded in paraffin as described (Marazziti et al., 2013).

### Potassium iodine (Lugol) staining

After dissection, murine brains were washed with PBS, fixed in 4% PFA for 30 min and transferred into 0.1 N (v/v) Lugol solution (Sigma, 32922). The samples were left in Lugol solution for 2 weeks at room temperature in a glass container in the dark. After this procedure, the samples were fitted in the narrow plastic column and placed in the microCT machine holder.

### MicroCT imaging

MicroCT imaging was performed as described in Ermakova et al. (2018). In brief, the 3D raw data were acquired by Skyscan 1172G (Bruker, Kontich, Belgium) using an L7901-20 Microfocus X-ray Source (Hamamatsu) with a camera pixel size of 9 µm, binning 2K. The X-ray tube voltage range was set at 39 kV, while the X-ray tube current was set at 240 µA (9 W). In all presented experiments, image acquisition was performed without filter. The samples were rotated by 180° during the volume acquisition. Reconstructions of the acquired 2D images in volume images were performed using built-in NRecon Skyscan reconstruction software (version: 1.6.6.0; Bruker). The 3D images were visualized using 3D Visualization Software CTvox v 2.5 (Bruker) to the volume rendering views and movies. Preparation of the images for presentation was performed using ImageJ software.

### Volumetric analysis

The volume measurements were performed using Bruker MicroCT-Analyser Version 1.13 software. Specific regions of the brain were manually defined as a region of interest (ROI) in every 2D section acquired by microCT, using the Allen Brain Atlas for guidance to accurately identify and segment each brain in a specific volume of interest (VOI). Separate data sets containing only the VOI region were created and used for automated volume measurements.

### Light microscopy

X-gal/FeCN-stained brains, embedded in paraffin, were sectioned at 16 µm and stained with Eosin Y 1% aqueous solution (Bio-Optica, 05-M10002). Images were obtained with the stereomicroscope MZ12 (Leica) equipped with color camera Leica DC500.

### Sperm preparation

At 6 weeks, *Ccdc151*-knockout males and wild-type littermates were sacrificed, and epididymis and vas deferenses were dissected as described in Behringer et al. (2014). Sperm was collected on a Petri dish in PBS media. Sperm counting and motility analysis was performed as described (La Sala et al., 2015).

### Induction of *ROSA26ERT2-Cre* recombinase expression by tamoxifen

Tamoxifen (Sigma, T5648) dissolved in corn oil (Sigma, C8267) at 10 mg/ml was administered to 4-month-old males at 1 mg per day by intraperitoneal injection for 5 consecutive days. Analysis of spermatogenesis was performed at 3 month post-induction of gene ablation.

## Supplementary Material

Supplementary information

## References

[DMM038489C1] Afzelius B. A. (2004). Cilia-related diseases. *J. Pathol.* 204, 470-477. 10.1002/path.1652 15495266 PMC7167937

[DMM038489C2] Alsaadi M. M., Erzurumluoglu A. M., Rodriguez S., Guthrie P. A., Gaunt T. R., Omar H. Z., Mubarak M., Alharbi K. K., Al-Rikabi A. C. and Day I. N. (2014). Nonsense mutation in coiled-coil domain containing 151 gene (CCDC151) causes primary ciliary dyskinesia. *Hum. Mutat.* 35, 1446-1448. 10.1002/humu.22698 25224326 PMC4489323

[DMM038489C3] Al-Shroof M., Karnik A. M., Karnik A. A., Longshore J., Sliman N. A. and Khan F. A. (2001). Ciliary dyskinesia associated with hydrocephalus and mental retardation in a Jordanian family. *Mayo Clin. Proc.* 76, 1219-1224. 10.4065/76.12.1219 11761503

[DMM038489C4] Behan L., Dimitrov B. D., Kuehni C. E., Hogg C., Carroll M., Evans H. J., Goutaki M., Harris A., Packham S., Walker W. T. et al. (2016). PICADAR: a diagnostic predictive tool for primary ciliary dyskinesia. *Eur. Respir. J.* 47, 1103-1112. 10.1183/13993003.01551-2015 26917608 PMC4819882

[DMM038489C5] Behringer R., Gertsenstein M. and Vinterstein K. (2014). *Manipulating of Mouse Embryo A Laboratory Manual*, 4the edn Edited by Cold Spring Harbor Laboratory.

[DMM038489C6] Ben Khelifa M., Coutton C., Zouari R., Karaouzène T., Rendu J., Bidart M., Yassine S., Pierre V., Delaroche J., Hennebicq S. (2014). Mutations in DNAH1, which encodes an inner arm heavy chain dynein, lead to male infertility from multiple morphological abnormalities of the sperm flagella. *Am. J. Hum. Genet.* 94, 95-104. 10.1016/j.ajhg.2013.11.017 24360805 PMC3882734

[DMM038489C7] Bustamante-Marin X. M. and Ostrowski L. E. (2017). Cilia and mucociliary clearance. *Cold Spring Harb. Perspect Biol.* 9 10.1101/cshperspect.a028241 PMC5378048 27864314

[DMM038489C8] Crews L., Wyss-Coray T. and Masliah E. (2004). Insights into the pathogenesis of hydrocephalus from transgenic and experimental animal models. *Brain Pathol.* 14, 312-316. 10.1111/j.1750-3639.2004.tb00070.x 15446587 PMC8095739

[DMM038489C9] De Santi M. M., Magni A., Valletta E. A., Gardi C. and Lungarella G. (1990). Hydrocephalus, bronchiectasis and ciliary aplasia. *Arch. Dis. Child.* 65, 543-544. 10.1136/adc.65.5.543 2357097 PMC1792142

[DMM038489C10] Eliasson R., Mossberg B., Camner P. and Afzelius B. A. (1977). The immotile-cilia syndrome. A congenital ciliary abnormality as an etiologic factor in chronic airway infections and male sterility. *N. Engl. J. Med.* 297, 1-6. 10.1056/NEJM197707072970101 301245

[DMM038489C11] Ermakova O., Orsini T., Gambadoro A., Chiani F. and Tocchini-Valentini G. P. (2018). Three-dimensional microCT imaging of murine embryonic development from immediate post-implantation to organogenesis: application for phenotyping analysis of early embryonic lethality in mutant animals. *Mamm. Genome* 29, 245-259. 10.1007/s00335-017-9723-6 29170794 PMC5887010

[DMM038489C12] Fassad M. R., Shoemark A., Legendre M., Hirst R. A., Koll F., le Borgne P., Louis B., Daudvohra F., Patel M. P., Thomas L. et al. (2018). Mutations in outer dynein arm heavy chain DNAH9 cause motile cilia defects and situs inversus. *Am. J. Hum. Genet.* 103, 984-994. 10.1016/j.ajhg.2018.10.016 30471717 PMC6288320

[DMM038489C13] Feldner A., Adam M. G., Tetzlaff F., Moll I., Komljenovic D., Sahm F., Bäuerle T., Ishikawa H., Schroten H., Korff T. et al. (2017). Loss of Mpdz impairs ependymal cell integrity leading to perinatal-onset hydrocephalus in mice. *EMBO Mol. Med.* 9, 890-905. 10.15252/emmm.201606430 28500065 PMC5494508

[DMM038489C14] Fliegauf M., Benzing T. and Omran H. (2007). When cilia go bad: cilia defects and ciliopathies. *Nat. Rev. Mol. Cell Biol.* 8, 880-893. 10.1038/nrm2278 17955020

[DMM038489C15] Flinn R., Evans C. C. and Lee L. (2014). Strain-dependent brain defects in mouse models of primary ciliary dyskinesia with mutations in Pcdp1 and Spef2. *Neuroscience* 277, 552-567. 10.1016/j.neuroscience.2014.07.029 25073043 PMC4164581

[DMM038489C16] Furey C. G., Choi J., Jin S. C., Zeng X., Timberlake A. T., Nelson-Williams C., Mansuri M. S., Lu Q., Duran D., Panchagnula S. et al. (2018). De Novo mutation in genes regulating neural stem cell fate in human congenital hydrocephalus. *Neuron* 99, 302-314. 10.1016/j.neuron.2018.06.019 29983323 PMC7839075

[DMM038489C17] Goutaki M., Meier A. B., Halbeisen F. S., Lucas J. S., Dell S. D., Maurer E., Spycher B. D. and Kuehni C. E. (2016). Clinical manifestations in primary ciliary dyskinesia: systematic review and meta-analysis. *Eur. Respir. J.* 48, 1081-1095. 10.1183/13993003.00736-2016 27492829

[DMM038489C18] Goutaki M., Eich M. O., Halbeisen F. S., Barben J., Casaulta C., Clarenbach C., Hafen G., Latzin P., Regamey N., Lazor R. et al. (2019). The swiss primary ciliary dyskinesia registry: objectives, methods and first results. *Swiss Med. Wkly.* 149, w20004 10.1101/450700 30691261

[DMM038489C19] Hjeij R., Onoufriadis A., Watson C. M., Slagle C. E., Klena N. T., Dougherty G. W., Kurkowiak M., Loges N. T., Diggle C. P., Morante N. F. et al. (2014). CCDC151 mutations cause primary ciliary dyskinesia by disruption of the outer dynein arm docking complex formation. *Am. J. Hum. Genet.* 95, 257-274. 10.1016/j.ajhg.2014.08.005 25192045 PMC4157146

[DMM038489C20] Horani A., Ferkol T. W., Dutcher S. K. and Brody S. L. (2016). Genetics and biology of primary ciliary dyskinesia. *Paediatr. Respir. Rev.* 18, 18-24. 10.1016/j.prrv.2015.09.001 26476603 PMC4864047

[DMM038489C21] Ibañez-Tallon I., Pagenstecher A., Fliegauf M., Olbrich H., Kispert A., Ketelsen U. P., North A., Heintz N. and Omran H. (2004). Dysfunction of axonemal dynein heavy chain Mdnah5 inhibits ependymal flow and reveals a novel mechanism for hydrocephalus formation. *Hum. Mol. Genet.* 13, 2133-2141. 10.1093/hmg/ddh219 15269178

[DMM038489C22] Ichioka K., Kohei N., Okubo K., Nishiyama H. and Terai A. (2006). Obstructive azoospermia associated with chronic sinopulmonary infection and situs inversus totalis. *Urology* 68, 204.e5-7 10.1016/j.urology.2006.01.072 16850538

[DMM038489C23] Jabourian Z., Lublin F. D., Adler A., Gonzales C., Northrup B. and Zwillenberg D. (1986). Hydrocephalus in Kartagener's syndrome. *Ear Nose Throat J.* 65, 469-472. 3490960

[DMM038489C24] Jerber J., Baas D., Soulavie F., Chhin B., Cortier E., Vesque C., Thomas J. and Durand B. (2014). The coiled-coil domain containing protein CCDC151 is required for the function of IFT-dependent motile cilia in animals. *Hum. Mol. Genet.* 23, 563-577. 10.1093/hmg/ddt445 24067530

[DMM038489C25] Jiménez A. J., Tomé M., Páez P., Wagner C., Rodríguez S., Fernández-Llebrez P., Rodríguez E. M. and Pérez-Fígares J. M. (2001). A programmed ependymal denudation precedes congenital hydrocephalus in the hyh mutant mouse. *J. Neuropathol. Exp. Neurol.* 60, 1105-1119. 10.1093/jnen/60.11.1105 11706940

[DMM038489C26] Knowles M. R., Daniels L. A., Davis S. D., Zariwala M. A. and Leigh M. W. (2013). Primary ciliary dyskinesia. Recent advances in diagnostics, genetics, and characterization of clinical disease. *Am. J. Respir. Crit. Care. Med.* 188, 913-922. 10.1164/rccm.201301-0059CI 23796196 PMC3826280

[DMM038489C27] Knowles M. R., Zariwala M. and Leigh M. (2016). Primary ciliary dyskinesia. *Clin. Chest Med.* 37, 449-461. 10.1016/j.ccm.2016.04.008 27514592 PMC4988337

[DMM038489C28] Kuehni C. E. and Lucas J. S. (2016). Toward an earlier diagnosis of primary ciliary dyskinesia. Which patients should undergo detailed diagnostic testing? *Ann. Am. Thorac. Soc.* 13, 1239-1243. 10.1513/AnnalsATS.201605-331PS 27258773

[DMM038489C29] Lang J. (1992). Topographic anatomy of performed intracranial spaces. *Acta Neurochir. Suppl. (Wien)* 54, 1-10. 10.1007/978-3-7091-6687-1_1 1595402

[DMM038489C30] La Sala G., Marazziti D., Di Pietro C., Golini E., Matteoni R. and Tocchini-Valentini G. P. (2015). Modulation of Dhh signaling and altered Sertoli cell function in mice lacking the GPR37-prosaposin receptor. *FASEB J.* 29, 2059-2069. 10.1096/fj.14-269209 25609427

[DMM038489C31] Lee L. (2013). Riding the wave of ependymal cilia: genetic susceptibility to hydrocephalus in primary ciliary dyskinesia. *J. Neurosci. Res.* 91, 1117-1132. 10.1002/jnr.23238 23686703

[DMM038489C32] Li Y., Klena N. T., Gabriel G. C., Liu X., Kim A. J., Lemke K., Chen Y., Chatterjee B., Devine W., Damerla R. R. et al. (2015). Global genetic analysis in mice unveils central role for cilia in congenital heart disease. *Nature* 521, 520-524. 10.1038/nature14269 25807483 PMC4617540

[DMM038489C33] Lin A. E., Krikov S., Riehle-Colarusso T., Frías J. L., Belmont J., Anderka M., Geva T., Getz K. D, Botto L. D. and National Birth Defects Prevention Study (2014). Laterality defects in the national birth defects prevention study (1998-2007): birth prevalence and descriptive epidemiology. *Am. J. Med. Genet.* 164A, 2581-2591. 10.1002/ajmg.a.36695 25099286 PMC4462240

[DMM038489C34] Lodish H., Berk A., Zipursky L. S., Matsudaira P., Baltimore D. and Darnell J. (2000). Cilia and flagella: structure and movement. In *Molecular Cell Biology*, 4th edn New York: W. H. Freeman and Co.

[DMM038489C35] Marazziti D., Di Pietro C., Golini E., Mandillo S., La Sala G., Matteoni R. and Tocchini-Valentini G. P. (2013). Precocious cerebellum development and improved motor functions in mice lacking the astrocyte cilium-, patched 1-associated Gpr37l1 receptor. *Proc. Natl. Acad. Sci. USA* 110, 16486-16491. 10.1073/pnas.1314819110 24062445 PMC3799331

[DMM038489C36] McAllister J. P. II (2012). Pathophysiology of congenital and neonatal hydrocephalus. *Semin. Fetal. Neonatal. Med.* 17, 285-294. 10.1016/j.siny.2012.06.004 22800608

[DMM038489C37] McKenzie C. W., Preston C. C., Finn R., Eyster K. M., Faustino R. S. and Lee L. (2018). Strain-specific differences in brain gene expression in a hydrocephalic mouse model with motile cilia dysfunction. *Sci. Rep.* 8, 13370 10.1038/s41598-018-31743-5 30190587 PMC6127338

[DMM038489C38] Mirra V., Werner C. and Santamaria F. (2017). Primary ciliary dyskinesia: an update on clinical aspects, genetics, diagnosis, and future treatment strategies. *Front. Pediatr.* 5, 135 10.3389/fped.2017.00135 28649564 PMC5465251

[DMM038489C39] Mitchison H. M. and Valente E. M. (2017). Motile and non-motile cilia in human pathology: from function to phenotypes. *J. Pathol.* 241, 294-309. 10.1002/path.4843 27859258

[DMM038489C40] Munro N. C., Currie D. C., Lindsay K. S., Ryder T. A., Rutman A., Dewar A., Greenstone M. A., Hendry W. F. and Cole P. J. (1994). Fertility in men with primary ciliary dyskinesia presenting with respiratory infection. *Thorax* 49, 684-687. 10.1136/thx.49.7.684 8066563 PMC475057

[DMM038489C41] Papon J. F., Coste A. and Roudot-Thoraval F. (2010). A 20-year experience of electron microscopy in the diagnosis of primary ciliary dyskinesia. *Eur. Respir. J.* 35, 1057-1063. 10.1183/09031936.00046209 19840971

[DMM038489C42] Park R., Moon U. Y., Park J. Y., Hughes L. J., Johnson R. L., Cho S. H. and Kim S. (2016). Yap is required for ependymal integrity and is suppressed in LPA-induced hydrocephalus. *Nat. Commun.* 7, 10329 10.1038/ncomms10329 26754915 PMC4729961

[DMM038489C43] Perez-Figares J. M., Jimenez A. J. and Rodriguez E. M. (2001). Subcommissural organ, cerebrospinal fluid circulation, and hydrocephalus Microsc. *Res. Tech.* 52, 591-607. 10.1002/1097-0029(20010301)52:5 11241868

[DMM038489C44] Picco P., Leveratto L., Cama A., Vigliarolo M. A., Levato G. L., Gattorno M., Zammarchi E. and Donati M. A. (1993). Immotile cilia syndrome associated with hydrocephalus and precocious puberty. A case report. *Eur. J. Pediatr. Surg.* 3, 20-21. 8130140

[DMM038489C45] Sampaio P., Ferreira R. R., Guerrero A., Pintado P., Tavares B., Amaro J., Smith A. A., Montenegro-Johnson T., Smith D. J. and Lopes S. S. (2014). Left-right organizer flow dynamics: how much cilia activity reliably yields laterality? *Dev. Cell* 29, 716-728. 10.1016/j.devcel.2014.04.030 24930722

[DMM038489C46] Shapiro A. J., Davis S. D., Ferkol T., Dell S. D., Rosenfeld M., Olivier K. N., Sagel S. D., Milla C., Zariwala M. A., Wolf W. et al. (2014). Genetic disorders of mucociliary clearance consortium. Laterality defects other than situs inversus totalis in primary ciliary dyskinesia: insights into situs ambiguus and heterotaxy. *Chest* 146, 1176-1186. 10.1378/chest.13-1704 24577564 PMC4219335

[DMM038489C47] Shoemark A., Dixon M., Corrin B. and Dewar A. (2012). Twenty-year review of quantitative transmission electron microscopy for the diagnosis of primary ciliary dyskinesia. *J. Clin. Pathol.* 65, 267-271. 10.1136/jclinpath-2011-200415 22135026

[DMM038489C48] Simon M. M., Greenaway S., White J. K., Fuchs H., Gailus-Durner V., Wells S., Sorg T., Wong K., Bedu E., Cartwright E. J. et al. (2013). A comparative phenotypic and genomic analysis of C57BL/6J and C57BL/6N mouse strains. *Genome Biol.* 14, R82 10.1186/gb-2013-14-7-r82 23902802 PMC4053787

[DMM038489C49] Skarnes W. C., Rosen B., West A. P., Koutsourakis M., Bushell W., Iyer V., Mujica A. O., Thomas M., Harrow J., Cox T. et al. (2011). A conditional knockout resource for the genome-wide study of mouse gene function. *Nature* 474, 337-342. 10.1038/nature10163 21677750 PMC3572410

[DMM038489C50] Tonosaki M., Itoh K., Umekage M., Kishimoto T., Yaoi T., Lemmon V. P. and Fushiki S. (2014). L1cam is crucial for cell locomotion and terminal translocation of the Soma in radial migration during murine corticogenesis. *PLoS ONE* 9, e86186 10.1371/journal.pone.0086186 24489698 PMC3904877

[DMM038489C51] Tully H. M. and Dobyns W. B. (2014). Infantile hydrocephalus: a review of epidemiology, classification and causes. *Eur. J. Med. Genet.* 57, 359-368. 10.1016/j.ejmg.2014.06.002 24932902 PMC4334358

[DMM038489C52] Viswanadha R., Sale W. S. and Porter M. E. (2017). Ciliary motility: regulation of axonemal dynein motors. *Cold Spring Harb Perspect Biol.* 9, a018325 10.1101/cshperspect.a018325 28765157 PMC5538414

[DMM038489C53] Wessels M. W., den Hollander N. S. and Willems P. J. (2003). Mild fetal cerebral ventriculomegaly as a prenatal sonographic marker for Kartagener syndrome. *Prenat. Diagn.* 23, 239-242. 10.1002/pd.551 12627427

[DMM038489C54] Yoshiba S. and Hamada H. (2014). Roles of cilia, fluid flow, and Ca2+ signaling in breaking of left-right symmetry. *Trends Genet.* 30, 10-17. 10.1016/j.tig.2013.09.001 24091059

[DMM038489C55] Yuan S., Liu Y., Peng H., Tang C., Hennig G. W., Wang Z., Wang L., Yu T., Klukovich R., Zhang Y. et al. (2019). Motile cilia of the male reproductive system require miR-34/miR-449 for development and function to generate luminal turbulence. *Proc. Natl. Acad. Sci. USA* 116, 3584-3593. 10.1073/pnas.1817018116 30659149 PMC6397547

[DMM038489C56] Zhang W., Li D., Wei S., Guo T., Wang J., Luo H., Yang Y. and Tan Z. (2019). Whole-exome sequencing identifies a novel CCDC151 mutation, c.325G>T (p.E109X), in a patient with primary ciliary dyskinesia and situs inversus. *J. Hum. Genet.* 64, 249-252. 10.1038/s10038-018-0540-x 30504913

