# Supplementary material for: Functional loss of Ccdc151 leads to hydrocephalus in a mouse model of primary ciliary dyskinesia
Source: Dis Model Mech. 2019 Aug 2;12(8):dmm038489. doi: 10.1242/dmm.038489 (PMC6737950; doi:10.1242/dmm.038489)
Supplement: Supplementary information [file dmm-12-038489-s1.pdf]

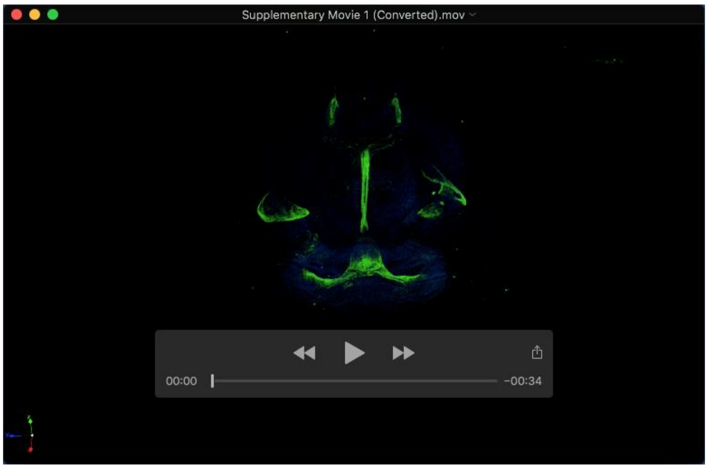

Movie 1. Whole-mount brains from Ccdc151-lacZ heterozygous animal were stained using X-gal/FeCN method and embedded in paraffin. Acquisition was performed with the image pixel size of 7,9 microns and beaning of 2K (image matrix 2000 x 1150 pixels). MicroCT derived RGB volume rendered model was build using the settings for CTvox analyzer (Bruker software) transfer function presented in the Fig. 3C.

Journal of Experimental Biology • Supplementary information

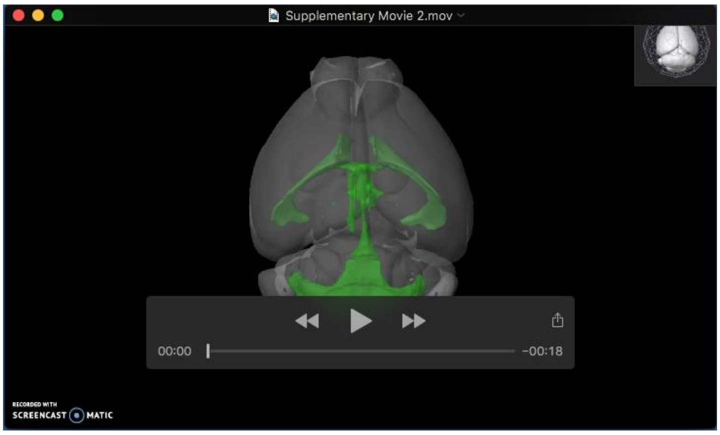

Movie 2. Virtually reconstructed 3D ventricular system of the murine brain, using Brain Explorer2 software defining only ventricular compartment of mouse brain (green color).

Journal of Experimental Biology • Supplementary information

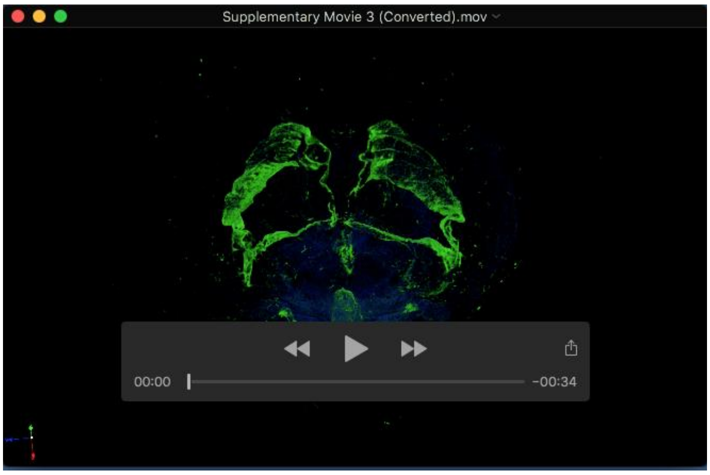

Movie 3. MicroCT derived RGB volume rendered model of the homozygous Ccdc151-/- (Ccdc151-lacZ) mouse brain stained with X-gal/FeCN method. Scan was performed at image pixel size of 7,9 microns and beaning of 2K. The settings for CTvox Bruker software transfer function are presented in the Fig. 4A.

Journal of Experimental Biology • Supplementary information

Movie 4

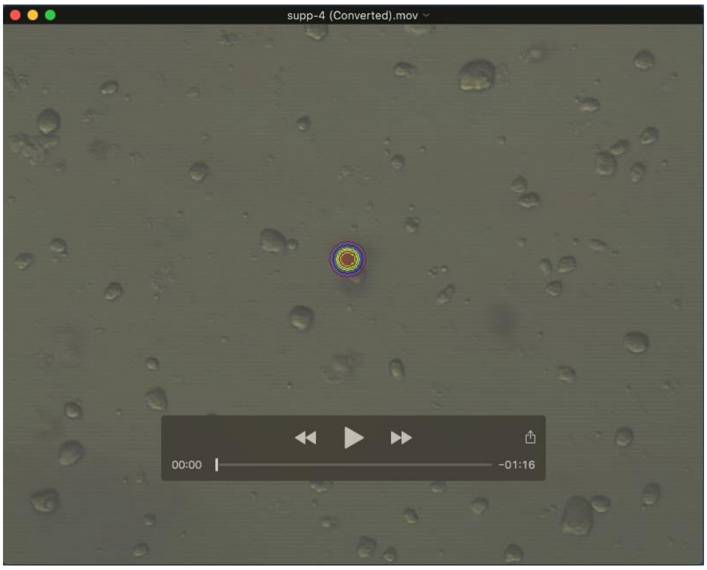

Movie 5

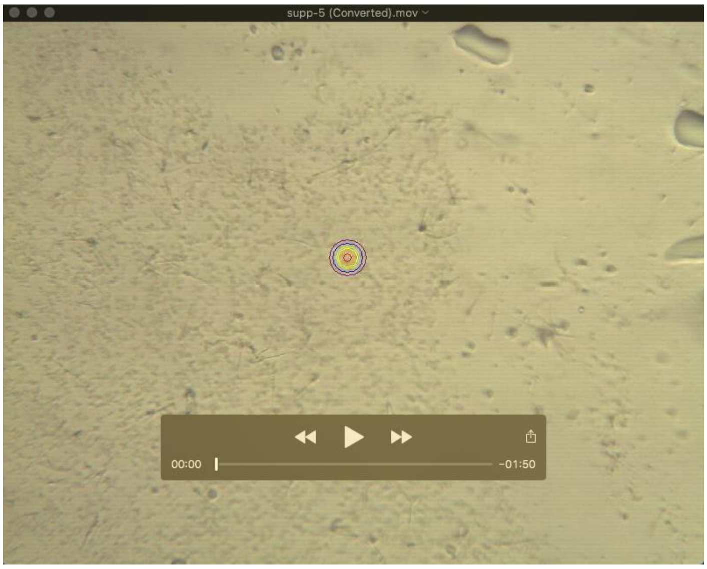

Movies 4,5. Surviving Ccdc151 knockout animals do not contain any sperm in epididymis and vas deferens. Sperm was collected from 6 weeks old males and deposited on a petri dish in PBS media and filmed at 20X magnification on an inverted microscope. Movie 4: No sperm was observed in preparation from the Ccdc151 homozygous knockout male; Movie 5: Sperm collected from the WT littermate.

Table S1. Summary of phenotypes observed in mouse models of PCD.

Click here to Download Table S1

Journal of Experimental Biology • Supplementary information
